# Early morning university classes are associated with impaired sleep and academic performance

**DOI:** 10.1038/s41562-023-01531-x

**Published:** 2023-02-20

**Authors:** Sing Chen Yeo, Clin K. Y. Lai, Jacinda Tan, Samantha Lim, Yuvan Chandramoghan, Teck Kiang Tan, Joshua J. Gooley

**Affiliations:** 1 Neuroscience and Behavioural Disorders Programme, Duke-NUS Medical School, Singapore, Singapore; 2 Institute for Applied Learning Sciences and Educational Technology, National University of Singapore, Singapore, Singapore

**Keywords:** Cognitive neuroscience, Human behaviour

## Abstract

Attending classes and sleeping well are important for students’ academic success. Here, we tested whether early morning classes are associated with lower attendance, shorter sleep and poorer academic achievement by analysing university students’ digital traces. Wi-Fi connection logs in 23,391 students revealed that lecture attendance was about ten percentage points lower for classes at 08:00 compared with later start times. Diurnal patterns of Learning Management System logins in 39,458 students and actigraphy data in 181 students demonstrated that nocturnal sleep was an hour shorter for early classes because students woke up earlier than usual. Analyses of grades in 33,818 students showed that the number of days per week they had morning classes was negatively correlated with grade point average. These findings suggest concerning associations between early morning classes and learning outcomes.

## Abstract (web summary)

Yeo et al. use digital data on student behaviour (Wi-Fi connections and Learning Management System logins) to examine associations between sleep and academic outcomes.

## Main

University students who regularly attend classes and sleep well are more likely to get good grades^1–4^. Attending classes increases students’ interactions with instructors and classmates and provides structured time for covering key learning points. Sleeping well is also important for optimizing cognitive performance and readiness to learn. Inadequate sleep impairs attention and memory processes^3,5–7^, which may prevent students from reaching their full learning potential in class (that is, presenteeism). Moreover, feeling tired and oversleeping are frequently cited as reasons why university students skip classes^8–11^. Effects of absenteeism and presenteeism on grades may have long-term consequences on students’ employment opportunities^12^, job performance ratings^13^ and salary^14^. Therefore, universities should adopt practices that improve students’ attendance rates and sleep behaviour to position them to succeed in the classroom and workforce.

Growing evidence indicates that early class start times can be detrimental for students’ sleep and daytime functioning. During adolescence and early adulthood, environmental and biological factors result in a delay in the preferred timing of sleep^15,16^. Hence, students who go to bed late and must wake up early for class have shorter nocturnal sleep^17^. The circadian drive for sleep may also reach its peak close to the time that students are expected to attend early morning classes. The combined effects of short sleep and circadian misalignment can lead to daytime sleepiness and impaired cognitive performance^18^. Delaying the start time of high schools has been shown to increase sleep duration and decrease sleepiness by allowing adolescents to sleep in longer^19–23^. However, there are mixed findings regarding the benefits of starting school later on absenteeism and academic outcomes. Meta-analyses and critical reviews have not found consistent evidence of improved attendance or grades^20,22,24^. Results were also shown to differ between schools after a district-wide delay in school start time^25^. Differences across studies and schools could be related to school characteristics, sampling bias or methods for evaluating attendance and academic achievement. Nonetheless, the large body of correlational and interventional work on school start times and sleep health in adolescents has led many school districts to delay their start times^26^.

Studies of school start times in adolescents may not be generalizable to university students who face environmental pressures that are markedly different compared with high school. The transition from high school to university is characterized by changes in students’ social and learning environments that can influence their sleep and learning behaviour. Many university students are living away from home for the first time and encounter new social contexts, demanding coursework and opportunities for late-night socializing^27^. The increased autonomy in how university students spend their time may lead to later bedtimes on school nights compared with when they were in high school^28^. University students also have a less-structured timetable in which the timing of their first class of the day can vary across the school week. This could lead to larger day-to-day changes in wake-up times and nocturnal sleep duration compared with high school students who usually have a fixed school start time^29^. Class attendance is also rarely monitored for lectures or seminars at universities, whereas attendance is compulsory and tracked in high schools. University students therefore have the freedom to skip classes and may decide, for example, to sleep instead of going to early morning classes. This, in turn, could impact students’ grades^30–34^.

Universities need scalable methods for evaluating the potential impact of class start times on students’ behaviour. Class scheduling practices are unlikely to change without university-wide evidence of a problem. Most studies on class start times and attendance in universities have been limited to convenience samples with small numbers of courses or students. Instructor-reported attendance was generally lower for earlier classes, but the underlying reasons (for example, oversleeping) were not evaluated^35–38^. Class attendance can be tracked on a much larger scale using mobile digital technologies that detect when students are present in the classroom. The Copenhagen Networks Study utilized smartphone sensors (Bluetooth and GPS signals) to estimate class attendance in about 1,000 university students over a 2-year period^2^, and a study at Tsinghua University used Wi-Fi connections and mobile application data to track class attendance in about 700 students over a 9-week period^39^. We also recently used Wi-Fi connection data to perform university-wide tracking of 24,000 students across different locations on campus including lecture halls and classrooms^40^. In contrast to previous studies, students were not required to use a study-specific software application on their smartphone, and our sample included all students who connected to the university Wi-Fi network. Given that nearly all students carry a Wi-Fi enabled device (for example, smartphone, laptop or tablet), Wi-Fi connection data could be used across the entire university to estimate class attendance without the need for active participation by students or instructors.

Earlier survey studies suggest that university students obtain more sleep when their morning classes start later (about 20 min more sleep when class starts 1 h later)^29,41^. However, objective evidence is lacking and traditional methods of collecting sleep–wake data (surveys and actigraphy) usually capture only a small fraction of the student population. The relationship between class start times and sleep–wake behaviour can potentially be determined at large scale by analysing university students’ diurnal pattern of digital traces. Students frequently interact with social media, smartphone and university digital learning platforms. Sustained periods of inactivity during the night indicate times when users are more likely to be sleeping. Consistent with the observation that sleep behaviour usually shifts later on free days compared with work/school days, a large-scale analysis of tweets across more than 1,500 US counties showed that the nocturnal period of low Twitter activity occurred later on weekends, public holidays and school holidays^42^. Other studies have shown that smartphone interactions (for example, touchscreen events) can be used to estimate sleep onset and offset because users often interact with their phone shortly before and after their nocturnal sleep^43,44^. However, these studies have not linked students’ diurnal pattern of digital traces to their school start times or learning outcomes. Recently, it was shown that students’ time-stamped logins on the university’s Learning Management System (LMS) can be used to profile their diurnal learning-directed behaviour^32^. Many universities use a LMS as the primary online platform for students to download course materials, submit assignments, complete quizzes and participate in class discussions. Similar to social media or smartphone activity, students’ interactions with the LMS represent a form of wake signal that can potentially be used to determine when sleep is likely to occur^32^. If LMS data can be shown to provide a reliable estimate of sleep timing, universities can use this information for measuring the aggregate impact of class start times on sleep behaviour of the entire student body.

The objective of our study was to test associations between class start times and attendance, sleep behaviour and academic performance at a large university. We developed scalable approaches for assessing students’ behaviour by analysing their digital traces in university-archived datasets. Wi-Fi connection data were used to estimate students’ lecture attendance rates and LMS data were used to estimate sleep opportunities. In parallel, an actigraphy study was conducted to validate and extend the findings. First, we tested the hypothesis that early morning classes are associated with lower Wi-Fi-confirmed attendance rates. Actigraphy data were used to determine whether students slept instead of attending early classes. Second, we tested the hypothesis that early morning classes are associated with earlier wake-up times and shorter sleep. This was assessed using LMS and actigraphy data sorted by students’ first class of the day. Third, we tested the hypothesis that morning classes are associated with lower course grades, and that students who have morning classes more days of the week have a lower grade point average.

## Results

### Class start times and students’ attendance

**Fig. 1:**
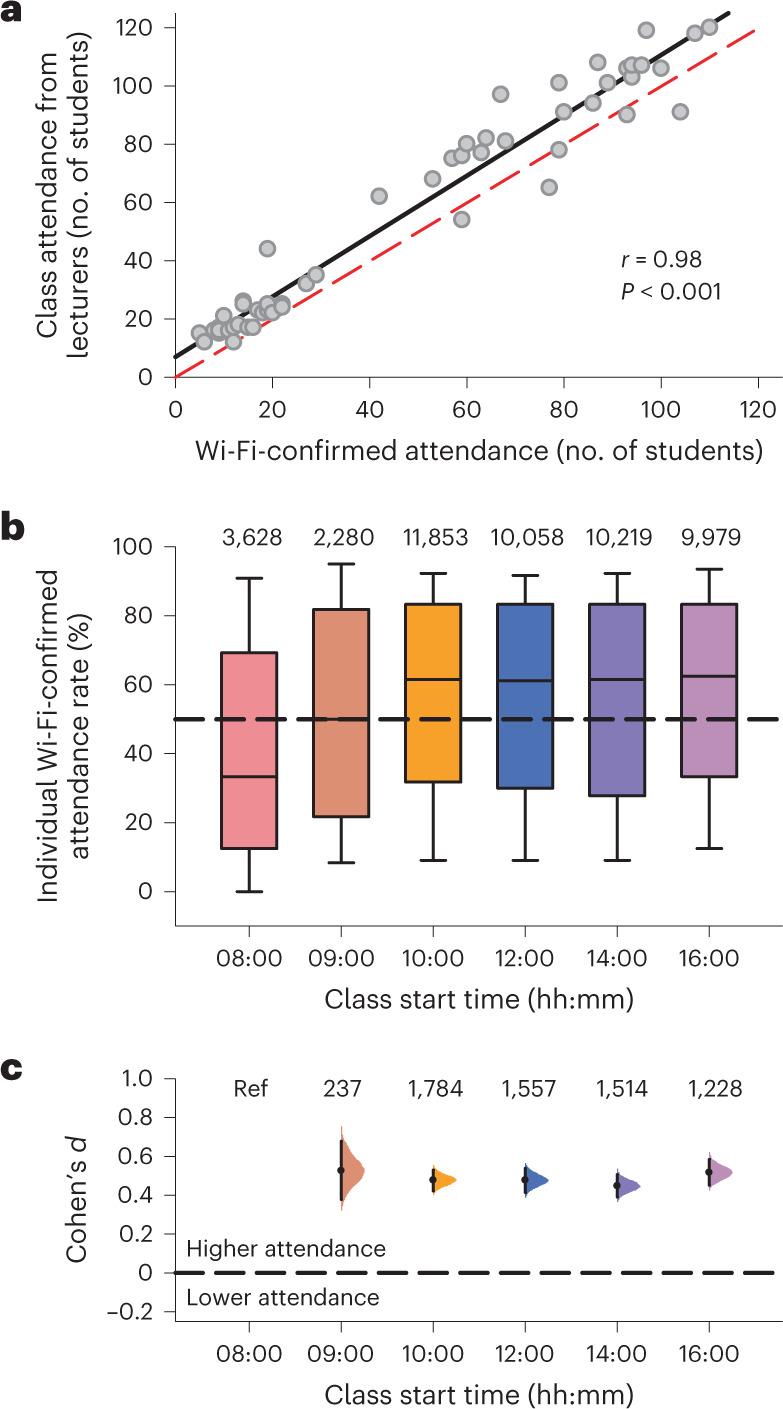
Wi-Fi-confirmed lecture attendance was lower for early morning classes. **a**, Instructor-reported attendance was strongly correlated with Wi-Fi-confirmed attendance (Pearson’s correlation analysis, two-tailed test: *r*(51) = 0.98, 95% CI = 0.97 to 0.99, *P* < 0.001). Each circle shows attendance data for an individual class session (53 class sessions across 13 different courses). The black trace shows the best-fit linear regression line and the red dashed trace is the unity line. **b**, Box plots show the distribution of individually determined Wi-Fi-confirmed attendance rates for different class start times in 23,391 unique students. Boxes show the median and interquartile range. Whiskers show the 10th and 90th percentiles. Sample sizes for each class start time are displayed at the top of each bar. **c**, Effect sizes (Cohen’s *d*) are shown for within-student comparisons of Wi-Fi-confirmed attendance rates. Effect sizes were determined for each class start time relative to 08:00. The number of students in each comparison is indicated at the top of the plot. The paired mean difference for each comparison is shown with 95% CI values and the bootstrap sampling distribution. Ref, reference category (08:00 class start time).

**Extended Data Fig. 1:**
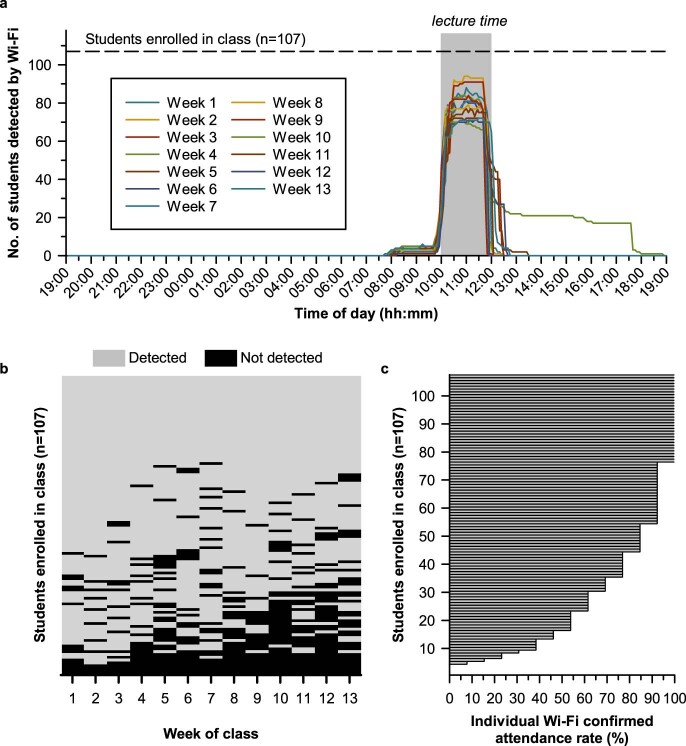
Method for using Wi-Fi connection data to estimate students’ class attendance. **a**–**c**, Wi-Fi confirmed attendance data are shown for a representative lecture course with a class enrolment of 107 students. The class took place weekly from 10:00 to 12:00 over 13 weeks. **a**, Daily time courses are shown for the number of enrolled students who connected to a Wi-Fi access point in the lecture hall where the class was conducted. The number of students detected by Wi-Fi increased sharply at the start of each class and then dropped at the end of class. There was one week when a group of about 20 students remained in the lecture hall for several hours after class. **b**, The binary heat map shows individually-determined Wi-Fi confirmed attendance (detected or not detected during class hours) across all 13 weeks of the semester. **c**, Wi-Fi confirmed attendance rates are shown for individual students in the course. Each row in panels **b** and **c** represents the same individual.

Students’ class attendance rates were estimated using time and location data from their Wi-Fi connection logs (Supplementary Table 1 and Extended Data Fig. 1). First, we showed that there was a strong linear correlation between instructor-reported attendance and Wi-Fi-confirmed attendance (53 class sessions; Pearson’s *r*(51) = 0.98, 95% confidence intervals (CI) = 0.97 to 0.99, *P* < 0.001), indicating that Wi-Fi connection data can be used as a relative indicator of class attendance (Fig. 1a). Next, this method was used to measure Wi-Fi-confirmed attendance rates for 23,391 unique students enroled across 337 large lecture courses (≥100 students enroled per course) with class start times ranging from 08:00 to 16:00 (Fig. 1b and Extended Data Fig. 1). The Wi-Fi-confirmed attendance rate for lecture classes at 08:00 was about ten percentage points lower compared with lecture classes that started at 10:00 or later, adjusting for course-level effects and demographic variables (difference in attendance rate relative to 08:00, Tukey’s test: 09:00, 7.6%, 95% CI = −1.9% to 17.1%, *P* = 0.64; 10:00, 11.1%, 95% CI = 3.9% to 18.3%, *P* = 0.032; 12:00, 11.0%, 95% CI = 3.6% to 18.4%, *P* = 0.043; 14:00, 10.8%, 95% CI = 3.5% to 18.2%, *P* = 0.047; 16:00, 11.3%, 95% CI = 4.0% to 18.7%, *P* = 0.032) (Supplementary Table 2a). In within-student comparisons of Wi-Fi-confirmed attendance rates, there was a medium effect size of starting class at 08:00 relative to other class start times (start time, Cohen’s *d*: 09:00, 0.53, 95% CI = 0.38 to 0.68; 10:00, 0.48, 95% CI = 0.42 to 0.53; 12:00, 0.48, 95% CI = 0.42 to 0.54; 14:00, 0.45, 95% CI = 0.39 to 0.51; 16:00, 0.52, 95% CI = 0.45 to 0.59) (Fig. 1c).

**Fig. 2:**
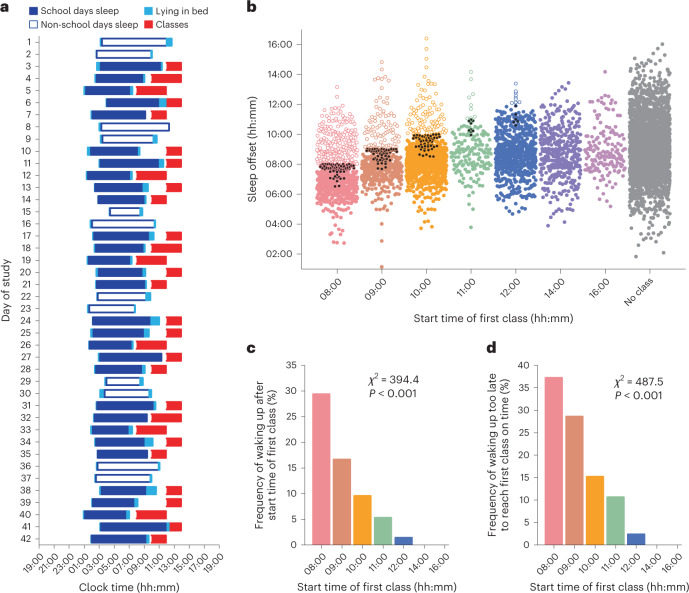
Students frequently slept past the start of morning classes. **a**, Sleep periods and scheduled classes are shown in a representative student who took part in a 6-week actigraphy study. **b**, Sleep offsets from 174 students are sorted by their first class of the day. Each circle corresponds to an individually determined sleep offset value. Open circles show instances when students woke up after the start of their class. Black circles show instances when students did not wake up early enough to reach class on time when their self-reported travel time was taken into account. **c**,**d**, The frequencies of (**c**) waking up after the start of class and (**d**) waking up too late to reach class on time were associated with class start times (two-sided chi-squared test: *χ*^2^(6) = 394.4, *P* < 0.001, Cramer’s *V* = 0.33, 95% CI = 0.31 to 0.37 and *χ*^2^(6) = 487.5*, P* < 0.001, Cramer’s *V* = 0.37, 95% CI = 0.35 to 0.40, respectively). Source data

Next, we examined whether students were absent from early morning classes because they were sleeping. Sleep behaviour was assessed in 181 students who took part in a 6-week actigraphy study during the school semester (Fig. 2a). Data for 6,546 sleep offset times were sorted by students’ first class of the day for individuals who provided information on their daily commute time to school (*n* = 174) (Fig. 2b). We then assessed the frequency of instances whereby students woke up after the start of their first class, or woke up before class but could not have reached class on time based on their self-reported travel time. The frequencies of sleeping past the start of class (Fig. 2c) and waking up too late to reach class on time (Fig. 2d) increased with earlier start times (two-sided chi-squared test: *χ*^2^(6) = 394.4, *P* < 0.001, Cramer’s *V* = 0.33, 95% CI = 0.31 to 0.37 and *χ*^2^(6) = 487.5, *P* < 0.001, Cramer’s *V* = 0.37, 95% CI = 0.35 to 0.40, respectively). Students did not wake up in time for nearly one-third of classes that took place at 08:00, whereas they rarely slept past the start of classes that began at noon or later. Next, we assessed whether students were more likely to take naps on days with early morning classes by analysing 336 actigraphy-verified naps. The frequency of napping was associated with class start time (two-sided chi-squared test: *χ*^2^(6) = 34.7, Cramer’s *V* = 0.10, 95% CI = 0.079 to 0.13, *P* < 0.001) and was highest when students’ first class of the day was in the morning (proportion of school days with naps by class start time: 08:00, 10.9%; 09:00, 10.3%; 10:00, 11.3%; 11:00, 10.3%; 12:00, 6.6%; 14:00, 3.5%; 16:00, 4.7%).

### Class start times and students’ sleep opportunities

**Fig. 3:**
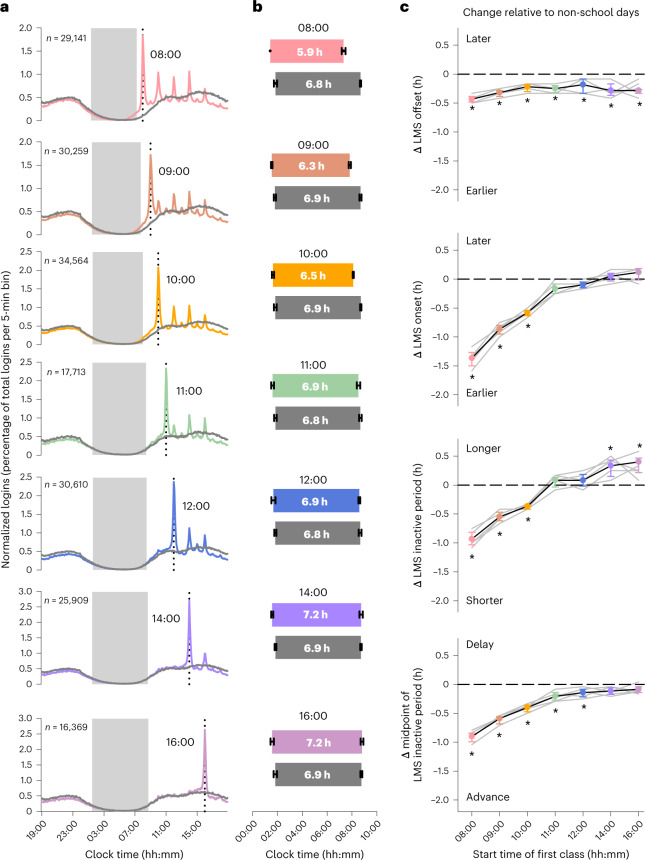
Earlier class start times were associated with shorter sleep opportunities. **a**, Diurnal time courses of LMS logins are shown in students whose data were sorted by their first class of the day. LMS logins were compiled from 39,458 unique students. Coloured traces show data for school days and grey traces show data for non-school days in the same group of students. The vertical dotted line in each plot shows the start time of students’ first class. The grey boxes show LMS inactive periods on school nights when login activity fell below threshold (Methods). **b**, LMS login offset and onset values are shown for different class start times (coloured bars) and compared with data for non-school days (grey bars). LMS parameters were determined separately for five different semesters and the mean ± 95% CI is shown for each set of five values. The average duration of the LMS inactive period is indicated in each bar. LMS parameters (offset, onset, inactive period, midpoint of inactive period) for each semester and school start time are provided in Supplementary Table 3. **c**, Changes in LMS login behaviour are shown for different class start times relative to non-school days. The paired mean differences were calculated separately for each semester and the 95% CIs are shown for each set of five values. Grey traces show results for individual semesters. Linear mixed-effects models were used to test for differences in LMS login parameters for each class start time relative to non-school days (two-tailed *t*-tests; Supplementary Table 2). Multiple comparisons were performed using Tukey’s test and asterisks show pairwise comparisons that reached statistical significance (*P* < 0.05).

**Extended Data Fig. 2:**
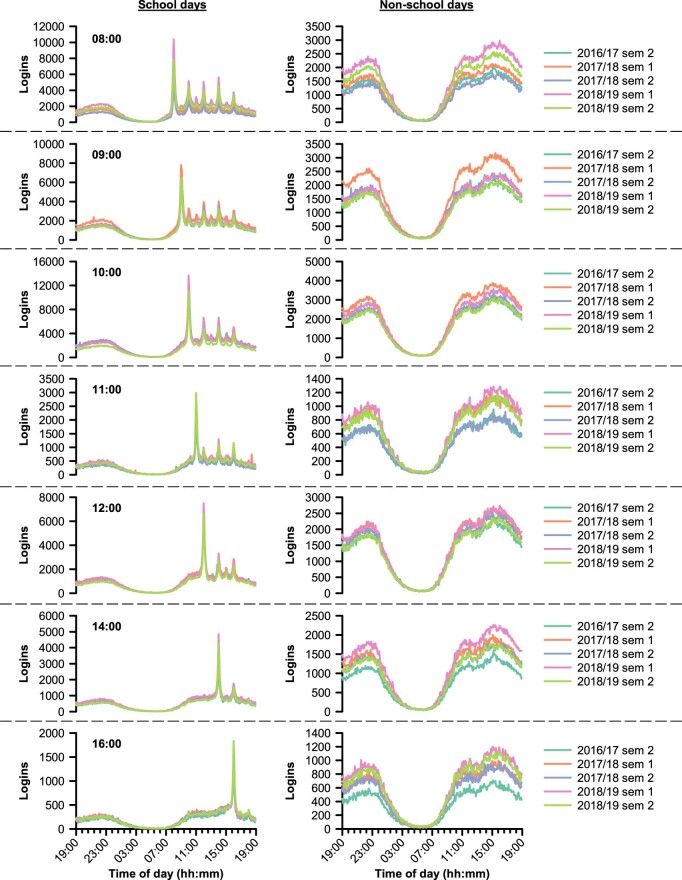
Daily time courses of Learning Management System (LMS) logins. Students’ LMS logins were compiled by time of day in 5-min bins over 5 different semesters. Data were sorted by students’ first class of the day (left column) and compared with non-school days (right column) in the same group of students. LMS logins were compiled from 39,458 unique students. The time courses of LMS logins were highly reproducible across semesters and academic years.

**Extended Data Fig. 3:**
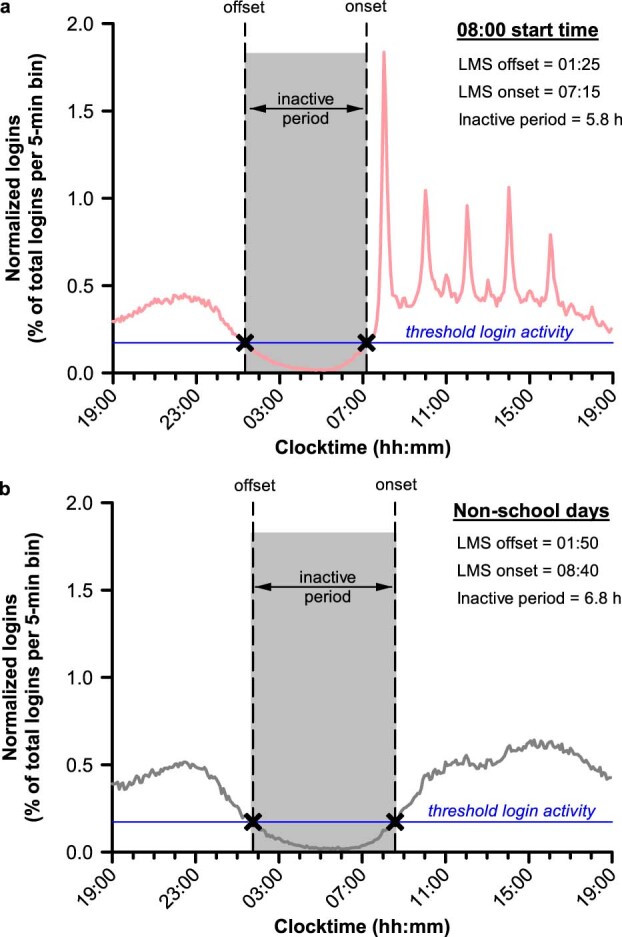
Method of deriving Learning Management System (LMS) login parameters. **a**,**b**, The diurnal distribution of LMS logins is shown on days that students had (**a**) classes starting at 08:00, and (**b**) no scheduled classes. Login data in both panels were compiled from the same group of students (*n* = 10,950) in the second semester of the 2018/19 academic year. Students’ LMS logins were summed by time of day in 5-min bins. The time series was normalized by dividing the number of logins in each 5-min bin by the total number of logins across all bins. LMS login parameters were derived using a threshold crossing method in which the LMS activity threshold was calculated as 50% of the average normalized number of logins per bin (1 bin / 288 bins × 0.5 = 0.001736 or 0.17%). The LMS login offset was defined as the clock time when the normalized number of logins dropped and remained below the threshold, and the LMS login onset was defined as the clock time when logins exceeded and stayed above the threshold. The blue line in each plot shows the LMS activity threshold, and the ‘X’ symbols show the downward and upward threshold crossings. The grey box indicates the LMS inactive period calculated from the LMS login offset to the LMS login onset.

Students’ opportunities for nocturnal sleep were investigated for different class start times by aggregating their LMS login data over five semesters (Supplementary Table 1). Given that students must be awake to log in to the LMS, we used this ‘wake signal’ to estimate when nocturnal sleep occurred. Diurnal time courses of LMS logins were constructed by compiling 17.4 million time-stamped logins from 39,458 students (Fig. 3a and Extended Data Fig. 2). LMS login data on school days were sorted by students’ first class of the day and compared with data on non-school days (Fig. 3a). The diurnal pattern of login activity on school days comprised a 24-h component with higher activity during the daytime and hourly peaks that corresponded to the timing of classes. The time courses of LMS logins for different class start times were highly reproducible across semesters (Extended Data Fig. 2). For each semester and class start time, the offset and onset of the aggregated LMS login rhythm were determined using a threshold crossing method (Methods and Extended Data Fig. 3) and the duration and midpoint of the LMS inactive period were measured between LMS offset and onset (Supplementary Table 3).

Next, we quantified associations of class start time (08:00, 09:00, 10:00, 11:00, 12:00, 14:00, 16:00 and non-school days) with LMS login behaviour. Relative to days with no classes, the LMS login offset occurred slightly earlier but did not vary much for different class start times (difference in LMS login offset relative to days with no classes, Tukey’s test: 08:00, −0.43 h, 95% CI = −0.52 h to −0.35 h, *P* < 0.001; 09:00, −0.32 h, 95% CI = −0.40 h to −0.23 h, *P* < 0.001; 10:00, −0.22 h, 95% CI = −0.30 h to −0.13 h, *P* = 0.001; 11:00, −0.25 h, 95% CI = −0.33 h to −0.17 h, *P* < 0.001; 12:00, −0.18 h, 95% CI = −0.27 h to −0.10 h, *P* = 0.009; 14:00, −0.28 h, 95% CI = −0.37 h to −0.21 h, *P* < 0.001; 16:00, −0.28 h, 95% CI = −0.37 h to −0.21 h, *P* < 0.001) (Fig. 3b,c and Supplementary Table 2b). By contrast, the LMS login onset tracked closely the start time of morning classes, with activity starting more than an hour earlier for classes at 08:00 relative to non-school days (difference in LMS login onset relative to days with no classes, Tukey’s test: 08:00, −1.37 h, 95% CI = −1.46 h to −1.27 h, *P* < 0.001; 09:00, −0.87 h, 95% CI = −0.96 h to −0.77 h, *P* < 0.001; 10:00, −0.58 h, 95% CI = −0.68 to −0.49, *P* = 0.001; 11:00, −0.17 h, 95% CI = −0.26 h to −0.07 h, *P* = 0.058; 12:00, −0.10 h, 95% CI = −0.19 h to −0.01 h, *P* = 0.55; 14:00, 0.05 h, 95% CI = −0.04 h to 0.14 h, *P* = 0.97; 16:00, 0.12 h, 95% CI = 0.02 h to 0.21 h, *P* = 0.36) (Fig. 3b,c and Supplementary Table 2c). Multiple comparison tests also showed that the LMS login onset was earlier for 08:00 classes compared with all other start times (difference in LMS login onset, Tukey’s test: 09:00, −0.50 h, 95% CI = −0.67 h to −0.33 h, *P* < 0.001; 10:00, −0.78 h, 95% CI = −0.95 h to −0.61 h, *P* < 0.001; 11:00, −1.20 h, 95% CI = −1.37 h to −1.03 h, *P* < 0.001; 12:00, −1.27 h, 95% CI = −1.44 h to −1.10 h, *P* < 0.001; 14:00, −1.42 h, 95% CI = −1.59 h to −1.25 h, *P* < 0.001; 16:00, −1.48 h, 95% CI = −1.65 h to −1.31 h, *P* < 0.001).

The LMS inactive period was about 1 h shorter when classes started at 08:00 compared with non-school days (difference in LMS inactive period relative to days with no classes, Tukey’s test: 08:00, −0.93 h, 95% CI = −1.05 h to −0.82 h, *P* < 0.001; 09:00, −0.55 h, 95% CI = −0.67 h to −0.43 h, *P* < 0.001; 10:00, −0.37 h, 95% CI = −0.48 to −0.25 h, *P* < 0.001; 11:00, 0.08 h, 95% CI = −0.03 h to 0.20 h, *P* = 0.90; 12:00, 0.08 h, 95% CI = −0.03 h to 0.20 h, *P* = 0.90; 14:00, 0.33 h, 95% CI = 0.22 h to 0.45 h, *P* < 0.001; 16:00, 0.40 h, 95% CI = 0.28 h to 0.52 h, *P* < 0.001) (Fig. 3b,c and Supplementary Table 2d). The LMS inactive period was also significantly shorter for 08:00 classes compared with all other start times (difference in LMS inactive period, Tukey’s test: 09:00, −0.38 h, 95% CI = −0.60 h to −0.17 h, *P* < 0.001; 10:00, −0.57 h, 95% CI = −0.78 h to −0.35 h, *P* < 0.001; 11:00, −1.02 h, 95% CI = −1.23 h to −0.80 h, *P* < 0.001; 12:00, −1.02 h, 95% CI = −1.23 h to −0.80 h, *P* < 0.001; 14:00, −1.27 h, 95% CI = −1.48 h to −1.05 h, *P* < 0.001; 16:00, −1.33 h, 95% CI = −1.55 h to −1.12 h, *P* < 0.001). The midpoint of the LMS inactive period occurred earlier for morning classes, with an advance of nearly 1 h on days when students had a class at 08:00 compared with non-school days (difference in midpoint of LMS inactive period relative to days with no classes, Tukey’s test: 08:00, −0.90 h, 95% CI = −0.96 h to −0.84 h, *P* < 0.001; 09:00, −0.59 h, 95% CI = −0.66 h to −0.53 h, *P* < 0.001; 10:00, −0.40 h, 95% CI = −0.46 h to −0.34 h, *P* < 0.001; 11:00, −0.21 h, 95% CI = −0.27 h to −0.14 h, *P* < 0.001; 12:00, −0.14 h, 95% CI = −0.21 h to −0.08 h, *P* = 0.010; 14:00, −0.12 h, 95% CI = −0.18 h to −0.05 h, *P* = 0.051; 16:00, −0.08 h, 95% CI = −0.15 h to −0.02 h, *P* = 0.31) (Fig. 3b,c and Supplementary Table 2e).

**Fig. 4:**
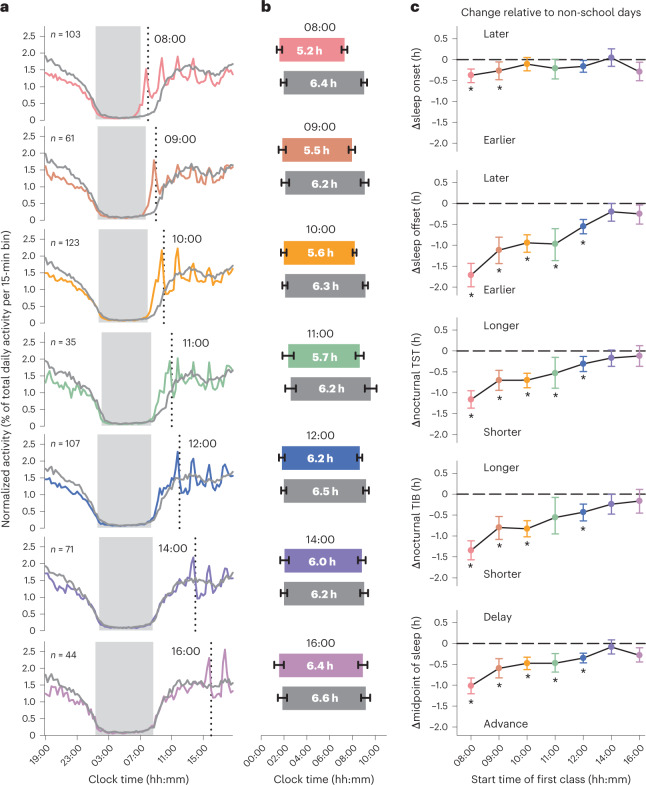
Earlier class start times were associated with shorter sleep. **a**, The averaged diurnal time courses of actigraphy-determined activity counts are shown in students (*n* = 181) whose data are sorted by their first class of the day. Coloured traces show data for school days and grey traces show data for non-school days in the same group of participants. The vertical dotted line in each plot shows the start time of students’ first class. The grey boxes show the average nocturnal sleep periods on days with classes. **b**, Sleep onset and sleep offset values (mean ± 95% CI) are shown for different class start times (coloured bars) and compared with data in the same students for non-school days (grey bars). The average nocturnal total sleep time is indicated in each bar. The distribution of individual values for sleep onset, sleep offset and nocturnal total sleep time are shown in Extended Data Fig. 4 (number of independent students for each class start time: 08:00, *n* = 103; 09:00, *n* = 61; 10:00, *n* = 123; 11:00, *n* = 35; 12:00, *n* = 107; 14:00, *n* = 71; 16:00, *n* = 44). **c**, In the same groups of students, changes in sleep behaviour are shown for different class start times relative to non-school days. The paired mean differences are shown with 95% CIs. Linear mixed-effects models were used to test for differences in sleep parameters for each class start time relative to non-school days (two-tailed *t*-tests; Supplementary Table 2). Multiple comparisons were performed using Tukey’s test and asterisks show pairwise comparisons that reached statistical significance (*P* < 0.05). TIB, time in bed for sleep; TST, total sleep time. Source data

**Extended Data Fig. 4:**
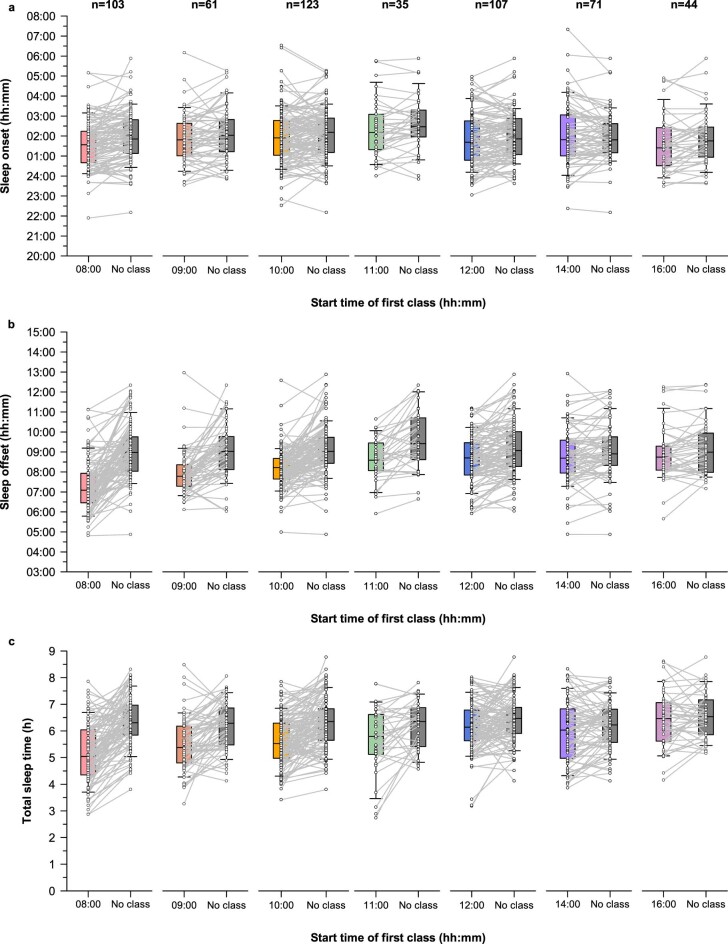
Sleep parameters sorted by students’ first class of the day. **a**–**c**, The distribution of individual values for actigraphically-determined (**a**) sleep onset, (**b**) sleep offset and (**c**) total sleep time are shown for different class start times (no. of independent students for each class start time: 08:00, n = 103; 09:00, n = 61; 10:00, n = 123; 11:00, n = 35; 12:00, n = 107; 14:00, n = 71; 16:00, n = 44). Circles indicate the median sleep value for an individual student for a given class start time. Gray lines connect data for the same individual to illustrate within-participant differences for school days versus non-school days. Box plots show the distribution of sleep values in each group of students for a given class start time. Boxes show the median and interquartile range. Whiskers show the 10^th^ and 90^th^ percentiles.

We validated findings for LMS login behaviour with actigraphy data collected from 181 undergraduates (Fig. 4 and Supplementary Table 4). The diurnal time courses of activity counts (wrist movements) closely resembled findings for the LMS login rhythm (Fig. 4a). Relative to non-school days, sleep onset occurred slightly earlier for classes that started at 08:00 or 09:00 but there was little variation across class start times, adjusting for demographic variables (difference in sleep onset relative to days with no classes, Tukey’s test: 08:00, −0.38 h, 95% CI = −0.49 h to −0.28 h, *P* < 0.001; 09:00, −0.22 h, 95% CI = −0.36 h to −0.09 h, *P* = 0.025; 10:00, −0.12 h, 95% CI = −0.22 h to −0.03 h, *P* = 0.19; 11:00, −0.18 h, 95% CI = −0.38 h to 0.03 h, *P* = 0.68; 12:00, −0.14 h, 95% CI = −0.25 h to −0.03 h, *P* = 0.22; 14:00, −0.07 h, 95% CI = −0.21 h to 0.07 h, *P* = 0.97; 16:00, −0.23 h, 95% CI = −0.43 h to −0.03 h, *P* = 0.32) (Fig. 4b,c, Extended Data Fig. 4a and Supplementary Table 2f). By contrast, sleep offset tracked the timing of students’ first class of the day, with larger advances for earlier morning classes (Cohen’s *d*, range = −1.29 to −0.75 for classes starting from 08:00 to 11:00; Supplementary Table 5). Students whose first class took place at 08:00 advanced their sleep offset by about 1.7 h relative to non-school days (difference in sleep offset relative to days with no classes, Tukey’s test: 08:00, −1.67 h, 95% CI = −1.78 h to −1.54 h, *P* < 0.001; 09:00, −1.05 h, 95% CI = −1.19 h to −0.90 h, *P* < 0.001; 10:00, −0.83 h, 95% CI = −0.94 h to −0.73 h, *P* < 0.001; 11:00, −0.59 h, 95% CI = −0.81 h to −0.36 h, *P* < 0.001; 12:00, −0.40 h, 95% CI = −0.52 h to −0.28 h, *P* < 0.001; 14:00, −0.23 h, 95% CI = −0.38 h to −0.07 h, *P* = 0.072; 16:00, −0.10 h, 95% CI = −0.32 h to 0.13 h, *P* = 0.99) (Fig. 4b,c, Extended Data Fig. 4b and Supplementary Table 2g). In addition, sleep offset was earlier for 08:00 classes compared with all other start times (difference in sleep offset, Tukey’s test: 09:00, −0.62 h, 95% CI = −0.89 h to −0.34 h, *P* < 0.001; 10:00, −0.83 h, 95% CI = −1.05 h to −0.61 h, *P* < 0.001; 11:00, −1.07 h, 95% CI = −1.45 h to −0.70 h, *P* < 0.001; 12:00, −1.26 h, 95% CI = −1.50 h to −1.02 h, *P* < 0.001; 14:00, −1.44 h, 95% CI = −1.71 h to −1.16 h, *P* < 0.001; 16:00, −1.57 h, 95% CI = −1.94 h to −1.19 h, *P* < 0.001).

Both nocturnal total sleep time and time in bed for sleep decreased with earlier class start times (Cohen’s *d*, range = −1.14 to −0.48 for classes starting from 08:00 to 11:00) (Supplementary Tables 4 and 5). Sleep was more than 1 h shorter in students with classes at 08:00 relative to non-school days, adjusting for covariates (difference in nocturnal total sleep time relative to days with no classes, Tukey’s test: 08:00, −1.16 h, 95% CI = −1.28 h to −1.05 h, *P* < 0.001; 09:00, −0.78 h, 95% CI = −0.93 h to −0.64 h, *P* < 0.001; 10:00, −0.65 h, 95% CI = −0.75 h to −0.54 h, *P* < 0.001; 11:00, −0.40 h, 95% CI = −0.62 h to −0.18 h, *P* = 0.010; 12:00, −0.23 h, 95% CI = −0.35 h to −0.10 h, *P* = 0.006; 14:00, −0.12 h, 95% CI = −0.27 h to 0.03 h, *P* = 0.76; 16:00, 0.11 h, 95% CI = −0.11 h to 0.33 h, *P* = 0.98) (Fig. 4b,c, Extended Data Fig. 4c and Supplementary Table 2h,i). The nocturnal sleep duration before 08:00 classes was shorter compared with all other start times (difference in nocturnal total sleep time for 08:00 classes relative to other start times, Tukey’s test: 09:00, −0.37 h, 95% CI = −0.64 h to −0.11 h, *P* < 0.001; 10:00, −0.51 h, 95% CI = −0.73 h to −0.30 h, *P* < 0.001; 11:00, −0.77 h, 95% CI = −1.13 h to −0.40 h, *P* < 0.001; 12:00, −0.94 h, 95% CI = −1.17 h to −0.70 h, *P* < 0.001; 14:00, −1.04 h, 95% CI = −1.31 h to −0.77 h, *P* < 0.001; 16:00, −1.27 h, 95% CI = −1.64 h to −0.90 h, *P* < 0.001). In addition, early class start times were associated with a greater advance in the midpoint of sleep (suggesting greater social jet lag) relative to non-school days (Cohen’s *d*, range = −0.89 to −0.40 for classes starting from 08:00 to 11:00) (Supplementary Table 5). Students’ midpoint of sleep occurred about 1 h earlier when they had a class at 08:00 on the following day, compared with days with no classes (difference in midpoint of sleep relative to days with no classes, Tukey’s test: 08:00, −1.02 h, 95% CI = −1.11 h to −0.93 h, *P* < 0.001; 09:00, −0.64 h, 95% CI = −0.75 h to −0.52 h, *P* < 0.001; 10:00, −0.48 h, 95% CI = −0.56 h to −0.40 h, *P* < 0.001; 11:00, −0.39 h, 95% CI = −0.56 h to −0.21 h, *P* < 0.001; 12:00, −0.27 h, 95% CI = −0.37 h to −0.17 h, *P* < 0.001; 14:00, −0.15 h, 95% CI = −0.27 h to −0.03 h, *P* = 0.23; 16:00, −0.17 h, 95% CI = −0.34 h to 0.01 h, *P* = 0.58) (Fig. 4b,c and Supplementary Table 2j).

**Extended Data Fig. 5:**
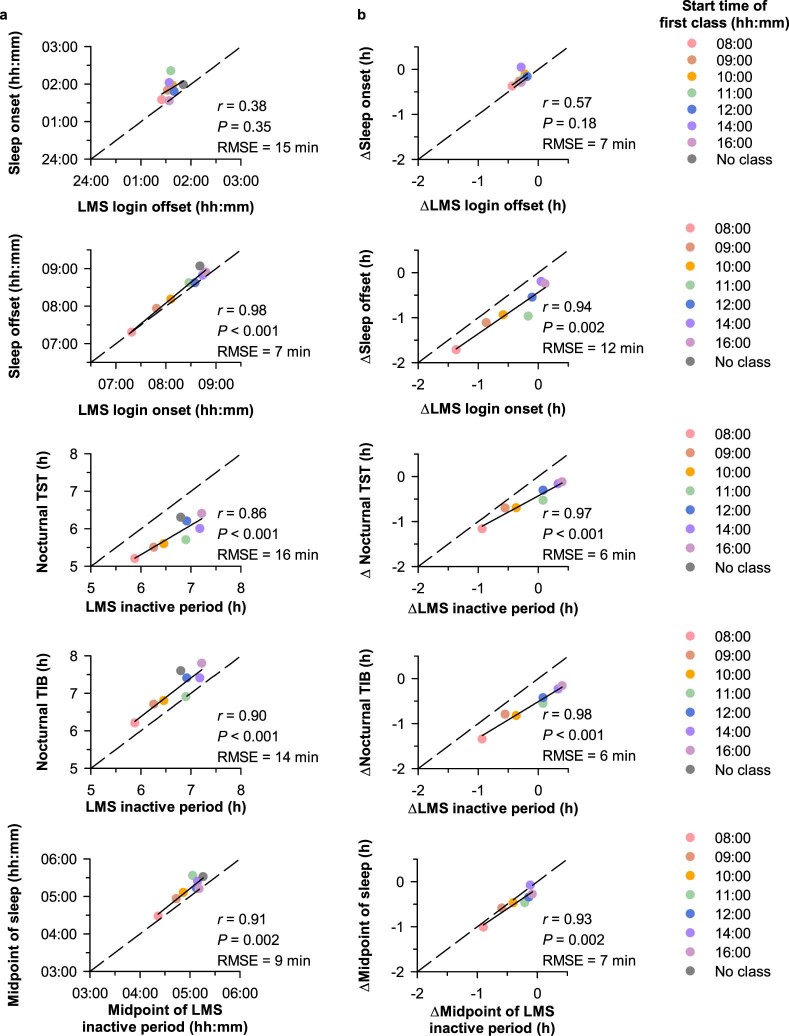
Correlations between Learning Management System (LMS) login parameters and actigraphy parameters. **a**,**b**, Scatter plots show the relationship between (**a**) LMS-derived parameters (averaged over 5 semesters) and actigraphy-derived sleep parameters (averaged across 181 students) for different class start times, and (**b**) changes in the timing and duration of these parameters on school days versus non-school days. Pearson’s correlation analysis (two-tailed test) was used to test the strength of the relationship between pairs of LMS and actigraphy variables (*r* = Pearson’s correlation coefficient; RMSE = root mean square error). Solid lines show the best-fit linear regression and dashed lines indicate the unity line. In panel **a**: sleep onset vs. LMS login offset, *r*(6)=0.38, 95% CI = -0.44 to 0.86, *P* = 0.35; sleep offset vs. LMS login onset, *r*(6)=0.98, 95% CI = 0.91 to 1.00, *P* < 0.001; nocturnal TST vs. LMS inactive period, *r*(6)=0.86, 95% CI = 0.39 to 0.97, *P* < 0.001; nocturnal TIB vs. LMS inactive period, *r*(6)=0.90, 95% CI = 0.52 to 0.98, *P* < 0.001; midpoint of sleep vs. midpoint of LMS inactive period, *r*(6)=0.91, 95% CI = 0.58 to 0.98, *P* = 0.002. In panel **b**: Δsleep onset vs. ΔLMS login offset, *r*(5)=0.57, 95% CI = -0.32 to 0.93, *P* = 0.18; Δsleep offset vs. ΔLMS login onset, *r*(5)=0.94, 95% CI = 0.64 to 0.99, *P* = 0.002; Δnocturnal TST vs. ΔLMS inactive period, *r*(5)=0.97, 95% CI = 0.79 to 1.00, *P* < 0.001; Δnocturnal TIB vs. ΔLMS inactive period, *r*(5)=0.98, 95% CI = 0.85 to 1.00, *P* < 0.001; Δmidpoint of sleep vs. Δmidpoint of LMS inactive period, *r*(5)=0.93, 95% CI = 0.60 to 0.99, *P* = 0.002. TIB = time in bed for sleep; TST = total sleep time.

Next, we quantified the degree to which LMS login parameters predicted sleep behaviour of the average student across different class start times by calculating Pearson’s correlation coefficient (*r*) and prediction error of the best-fit linear regression (root mean square error (r.m.s.e.)) (Extended Data Fig. 5a). As expected, there was no statistically significant correlation between LMS login offset and sleep onset because these parameters did not vary much across different class start times (*r*(6) = 0.38, 95% CI = −0.44 to 0.86, *P* = 0.35, r.m.s.e. = 15 min). However, the LMS login onset closely approximated the sleep offset and these measures were highly correlated (*r*(6) = 0.98, 95% CI = 0.91 to 1.00, *P* < 0.001, r.m.s.e. = 7 min). Strong correlations were also observed between the LMS inactive period and nocturnal total sleep time (*r*(6) = 0.86, 95% CI 0.39 to 0.97, *P* < 0.001, r.m.s.e. = 14 min), the LMS inactive period and nocturnal time in bed for sleep (*r*(6) = 0.90, 95% CI = 0.52 to 0.98, *P* < 0.001, r.m.s.e. = 16 min) and the midpoint of the LMS inactive period and the midpoint of sleep (*r*(6) = 0.91, 95% CI = 0.58 to 0.98, *P* = 0.002, r.m.s.e. = 9 min). Changes in the timing and duration of LMS and actigraphy parameters on school days relative to non-school days were also closely related (Extended Data Fig. 5b).

### Class start times and grades

**Fig. 5:**
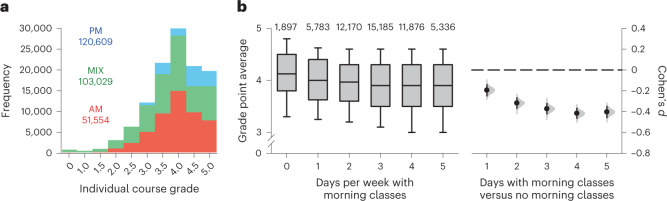
Students with morning classes on more days of the week had a lower grade point average. **a**, The distributions of grades are shown for courses with class sessions that took place exclusively in the morning (starting before 12:00), exclusively in the afternoon (starting at 12:00 or later) or in both the morning and afternoon. The number of morning-only (AM), afternoon-only (PM) and morning/afternoon (MIX) course grades is indicated on the plot. **b**, Box plots show the distribution of grade point average by the number of days per week that students had morning classes. Boxes show the median and interquartile range. Whiskers show the 10th and 90th percentiles. Sample sizes are displayed at the top of each bar. Effect sizes (Cohen’s *d*) with 95% CIs and the bootstrap sampling distributions are plotted for days with morning classes (1 to 5 days) versus having no morning classes. Data were compiled from 33,818 unique students over six semesters.

The relationship between class start time and grades was analysed in 33,818 students taking the same number of course credits (that is, equivalent workload expressed in time units) (Supplementary Table 1). Owing to heterogeneity in the timing of classes within courses (for example, a lecture and tutorial scheduled at different times for the same course), we categorized courses as occurring exclusively in the morning, exclusively in the afternoon or in both the morning and afternoon (Fig. 5a). Course grades were marginally higher for afternoon-only courses, but not mixed morning/afternoon courses, compared with morning-only courses (difference in grade point relative to morning-only courses, Tukey’s test: afternoon-only courses, 0.016, 95% CI = 0.006 to 0.026, *P* = 0.0042; mixed morning/afternoon courses, −0.001, 95% CI = −0.011 to 0.009, *P* = 0.80) (Supplementary Table 2k).

Next, we tested whether students with morning classes on more days of the week had a lower grade point average (for example, due to cumulative effects of lower attendance or shorter sleep on overall performance). Students with no morning classes had a higher grade point average than all other groups, adjusting for covariates (difference in grade point average relative to having no days with morning classes, Tukey’s test: 1 day per week, −0.069, 95% CI = −0.094 to −0.044, *P* < 0.001; 2 days per week, −0.103, 95% CI = −0.126 to −0.079, *P* < 0.001; 3 days per week, −0.117, 95% CI = −0.141 to –0.093, *P* < 0.001; 4 days per week, −0.141, 95% CI = −0.165 to −0.116, *P* < 0.001; 5 days per week, −0.146, 95% CI = −0.173 to −0.119, *P* < 0.001) (Fig. 5b and Supplementary Table 2l). Relative to students with no morning classes, having morning classes on one or two days of the week was associated with a small effect size for grade point average (Cohen’s *d* = −0.20, 95% CI = −0.25 to −0.14 and −0.32, 95% CI = −0.37 to −0.27, respectively), and having morning classes on three to five days of the week was associated with a medium effect size for grade point average (Cohen’s *d* = −0.37, 95% CI = −0.42 to −0.32 (three days), −0.42, 95% CI = −0.46 to −0.37 (four days) and −0.40, 95% CI = −0.46 to −0.35 (five days)) (Fig. 5b).

## Discussion

Our study showed that sleep behaviour and learning-related outcomes were associated with the time of day that university students had their first class. Many students may be forced to make one of two undesirable choices when faced with early class start times: sleep longer instead of attending class or wake up earlier to attend class. Wi-Fi-confirmed attendance rates were about ten percentage points lower in students taking classes at 08:00 compared with later class start times. Even though students frequently slept past the start of classes at 08:00, they still lost about 1 h of sleep on average compared with days with only afternoon classes or no classes. This was shown using LMS-derived sleep opportunities and confirmed by actigraphy. Students who had more days of morning classes in a week also had a lower grade point average. Our findings suggest that there might be cumulative negative effects of morning classes on absenteeism and presenteeism that lead to poorer academic achievement.

Our findings for Wi-Fi-confirmed attendance extend previous studies in university students demonstrating that instructor-reported attendance was generally lower earlier in the day^35–38^. Previous studies assessed attendance for only a few courses, whereas analysing students’ Wi-Fi digital traces made it possible to estimate class attendance at large scale; that is, across hundreds of courses with different start times. A previous study also used Wi-Fi connection data to track class attendance in several hundred university students, but their method of counting students was not verified by instructor-reported attendance and the sample was restricted to volunteers who were using the university’s mobile application software (Android users only). By combining students’ Wi-Fi connection logs with their course timetables, we were able to derive estimates of class attendance in more than 23,000 students without requiring their active participation. This method was used to show that lecture attendance was lowest for classes that started at 08:00. Moreover, we provided objective evidence that students often slept instead of going to early morning classes. Actigraphy data showed that students overslept for nearly one-third of lecture classes that started at 08:00. These results are consistent with survey studies of university students in which common reasons for absenteeism included lectures occurring too early, lack of sleep, feeling tired and oversleeping^8–11^.

We showed that students’ population behaviour of diurnal LMS login activity can be used to estimate nocturnal sleep opportunities. Previous studies applied a related approach in which low periods of Twitter or smartphone activity were used to estimate sleep behaviour^42,45,46^. These studies, which were not restricted to student populations, observed a delay in the nocturnal inactive period on weekends and holiday periods compared with weekdays, but they did not investigate the effects of different school/work start times. We sorted students’ LMS login data by their first class of the day and verified the results in an actigraphy study with several thousand nocturnal sleep recordings. The LMS login onset closely tracked students’ wake-up time for morning classes, resulting in a shorter LMS inactive period compared with afternoon start times or non-school days. Population-derived LMS login parameters were strongly associated with student-averaged actigraphy parameters with low prediction errors for wake-up time, total nocturnal sleep duration and midpoint of sleep (r.m.s.e. < 16 min for all comparisons). Both the LMS login offset and sleep onset showed little variation for different class start times. Together, these results showed that LMS login behaviour and sleep–wake behaviour covaried with students’ first class of the day.

We provided evidence that early morning classes may contribute to a university-wide sleep debt and circadian misalignment. Students went to bed at around the same time but woke up earlier to attend morning classes. Consequently, nocturnal sleep duration was shorter only on nights that preceded morning classes. Our results for objective sleep behaviour extend earlier work in undergraduates demonstrating that self-reported sleep duration was shorter when students’ first class of the day was earlier in the morning^29,41^. The midpoint of sleep also occurred earlier for morning classes, suggesting greater social jet lag. Similar to our results, a previous study found that undergraduates with classes only in the morning (07:00–12:00) had shorter sleep on weekdays and extended their sleep duration on weekends, whereas students with classes only in the afternoon (14:00–18:00) did not show a difference in sleep duration between weekdays and weekends^47^. Comparable results were obtained in adolescents attending schools with morning and afternoon/evening shifts, in which students with later class schedules obtained more sleep on school days and were less likely to extend their sleep on weekends^48–54^. We also found that the frequency of daytime naps was higher when students had morning classes, suggesting that students were sleepier compared with days with later class start times. Compensatory napping may contribute to reduced daytime light exposure and circadian desynchrony. These findings for sleep behaviour are consistent with models that predict early start times should be avoided for optimal cognitive performance in undergraduates^55^.

We found that course grades were statistically lower for courses held only in the morning versus only in the afternoon. However, the difference in grades was very small (one-hundredth of a grade point) and probably not meaningful. Previous studies found that grades were either lower in the morning compared with the afternoon^30–34^, or there were no differences across time of day^56^. At the university in our study, course grades are usually adjusted to meet a recommended grade distribution (that is, graded on a curve). By design, the average grade does not differ much between courses and may mask underlying performance differences between morning and afternoon courses. Students may have also chosen courses and start times that suited their personal preferences or schedules. For example, morning-type students may have been more likely to enrol in early morning classes, whereas late-type students may have been more likely to avoid them. Course characteristics (for example, course difficulty, grading criteria or teacher performance) may have also differed across the day and students’ performance in afternoon courses may have been influenced by whether they had classes scheduled earlier in the day^34^.

We showed that having morning classes on more days of the week was associated with poorer academic performance. In contrast to individual course grades, this analysis considered when students’ first class of the day took place. If morning classes have a cumulative negative impact on students’ attendance and sleep, performance should be lower for classes at other times of day. Consistent with this expectation, grade point average was highest for students with no morning classes and decreased when students had more days of morning classes. Similar to this result, a previous study found that students who had a first period course before 08:00 performed worse in all other courses taken on the same day, compared with students who did not have a first period course^34^. In our study, the effect size was comparable in magnitude to many student- and teacher-related interventions for improving academic achievement, including peer tutoring, integrated curriculum programmes, small group learning, computer-assisted instruction, intelligent tutoring systems, cooperative learning, gaming/simulations, student-centred teaching and bilingual programmes (Cohen’s *d* ranging from 0.35 to 0.55)^57^. Hence, future studies should assess whether reducing the number of days that university students have morning classes leads to meaningful improvements in learning.

In our study, there are limitations associated with using students’ digital traces to estimate learning-related behaviours. Although Wi-Fi connection data made it possible to estimate attendance rates in large numbers of students and courses, this method requires that students use a Wi-Fi enabled device that is actively scanning for wireless access points. Some students may have disabled Wi-Fi scanning on their devices or used a cellular data plan instead. Nonetheless, we found that Wi-Fi-confirmed attendance underestimated instructor-reported attendance by only a small amount. The main limitation of using LMS data for estimating sleep behaviour is that students typically interact with the LMS only a few times each day. Consequently, the LMS method we used cannot estimate day-to-day changes in sleep behaviour in individual students. In addition, students’ interactions with the LMS may be influenced by factors unrelated to sleep–wake behaviour; for example, personal preferences or social schedules. Our analysis approach was similar to a previous study of Twitter activity in which the daily time course of tweets on weekdays and weekends was determined by pooling data across many Twitter users^42^. By comparison, smartphone interactions may be better at estimating daily fluctuations in sleep behaviour^43,44^. Despite limitations associated with using LMS data, we showed that LMS logins accumulate in large numbers over time and can provide universities with an aggregate view of how their class scheduling practices may influence students’ sleep opportunities.

Future studies of university students should test the interventional effects of delaying the start of morning classes on sleep and learning behaviour. There is a strong theoretical basis for starting school later to improve students’ sleep and daytime functioning, but our study was observational and did not establish causality. Our findings were consistent with earlier interventional studies performed at high schools where delaying start times resulted in longer sleep durations and reduced daytime sleepiness^7,22,24,25,58^. The few studies that randomly allocated students to different school start times found that earlier start times were associated with shorter sleep and lower grades^34,48,59^. Similarly, university students who were randomly assigned to different class start times in courses with multiple sections had lower attendance and grades for classes that started at 08:00 or 09:00 compared with later in the day^30,35^. More studies are needed to assess whether starting classes later can result in sustained improvements in sleep behaviour and class attendance. Some students may gradually shift their bedtime later, hence reducing the benefit of starting classes later. Concurrent interventions to improve students’ self-regulation skills may be important for ensuring that students allocate enough time for sleep and attend classes. Such interventions could even be implemented using the LMS to provide feedback to students on how to improve their sleep and learning behaviour^60^.

In conclusion, our study suggests that universities should consider avoiding mandatory early morning classes. Although early classes are often scheduled to maximize use of resources (classroom space and faculty time spent on teaching) and to minimize scheduling conflicts for students and faculty, our results indicate that there may be a trade-off, whereby students are more likely to miss class, get less sleep and obtain a lower grade point average. Early classes could be scheduled later in the day if classrooms and lecture theatres are not being fully utilized, and making classrooms a shared resource across departments might open up time slots for more afternoon/evening courses to be conducted in parallel. To justify taking such actions, universities need scalable methods for assessing the impact of their class scheduling practices on students. Our study showed that archived digital traces that are routinely collected by universities can be used to assess relationships between class start times and students’ behaviour. In future studies, these approaches can be used to test the effectiveness of interventions for improving students’ class attendance, sleep and academic achievement.

## Methods

### Wi-Fi-confirmed attendance

Students’ Wi-Fi connection metadata were archived on the National University of Singapore (NUS) Institute for Applied Learning Sciences and Educational Technology (ALSET) Data Lake. Each time that a student’s Wi-Fi-enabled device associated with the NUS wireless network, the transmission data were logged. Data included the tokenized student identity, the anonymized media access control address used to identify the Wi-Fi-enabled device, the name and location descriptor of the Wi-Fi access point and the start and end time of each Wi-Fi connection. The campus wireless network at NUS comprises more than 6,500 Wi-Fi access points, including coverage of classrooms and lecture halls^40^. Students’ Wi-Fi connections at these locations were cross-referenced with their course timetables. These time and location data made it possible to identify students who connected to a Wi-Fi router in their classroom during class hours, thereby confirming their attendance.

The method of using Wi-Fi connection data to estimate class attendance was validated by collecting attendance data from course instructors. Attendance data were obtained for 53 class sessions across 13 different courses. Instructors who provided attendance data were recruited from a convenience sample of faculty involved in educational research. In each of the class sessions, we determined the number of enroled students with at least one Wi-Fi connection during class. The strength of the linear correlation between instructor-reported attendance and Wi-Fi-confirmed attendance was assessed using Pearson’s correlation analysis (two-sided test; SigmaPlot v.14.5, Systat Software, Inc.).

Wi-Fi-confirmed attendance was investigated over three semesters (2018/19 semester one, 2018/19 semester two and 2019/20 semester one) using all available data on the ALSET Data Lake before the COVID-19 pandemic. We decided to focus on large lecture courses because we expected that students would be more likely to skip these classes compared with other types of courses that are smaller and more interactive. Absenteeism is less likely to be noticed or tracked in larger lecture courses, and in-class participation is not a key element of most lecture courses. Hence, students may be more willing to skip these classes in favour of their preferred sleep/wake schedule or personal interests. Courses were considered for the analysis if: (1) they were categorized as a lecture course according to the NUS timetable, (2) they were held once per week, (3) they were held at least seven times over the 13-week semester (half-semester courses were excluded), (4) they lasted 2 h per session and (5) they had at least 100 students enroled in the course. The rationale for these criteria was to ensure that comparable types of courses were included in the analyses across different class start times. The weekly 2-h format is the most common for lecture courses at the university, and setting a cut-off of 100 students per course ensured that classes took place in one of the lecture halls. Among the 436 courses that met these criteria, 71 were excluded because of missing or incomplete Wi-Fi connection data or inconsistencies with the class timetable (for example, cancelled or rescheduled classes). The remaining 365 courses were sorted by their start time, and data were analysed only for those start times in which there were at least five courses per semester to ensure that we had sufficient data to make meaningful comparisons between class start times (08:00, 21 courses; 09:00, 18 courses; 10:00, 89 courses; 12:00, 67 courses; 14:00, 72 courses; and 16:00, 70 courses). The final dataset included 337 courses and 23,391 unique students. Demographic characteristics of students are provided in Supplementary Table 1. The average class size (number of students enroled in the course) in the dataset was 193 ± 73 students (mean ± s.d.) and class size did not differ between lecture start times (one-way analysis of variance: *F*(5,331) = 0.91, *P* = 0.476). The Wi-Fi-confirmed attendance rate for each student was determined in each of the 337 courses. In a given course, this was calculated as the number of lectures in which a student was detected by Wi-Fi, divided by the total number of lectures held during the semester (Extended Data Fig. 1).

### LMS data

Students’ interactions with the university’s LMS were analysed over five semesters (2016/17 semester two, 2017/18 semester one, 2017/18 semester two, 2018/19 semester one, 2018/19 semester two) using all available data on the ALSET Data Lake. The diurnal time courses of logins were analysed separately in each semester by sorting the data according to each student’s first class start time of the day. For a given semester and class start time, the total number of logins per 5-min bin was summed across all students starting from 19:00 on the previous evening until 19:00 in the evening of the day on which the class took place (288 epochs per day) (Supplementary Methods). Analyses were restricted to the most frequent class start times at NUS for which we also had sufficient actigraphy data to make comparisons (08:00, 09:00, 10:00, 11:00, 12:00, 14:00 and 16:00). Data were also analysed on non-school days, corresponding to weekends and weekdays with no scheduled classes. The aggregated time series data allowed us to compare students’ diurnal login behaviour by their first class of the day and relative to days with no classes. The dataset comprised 17.4 million logins from 39,458 students. Students’ demographic characteristics are provided for each semester in Supplementary Table 1.

The time courses of logins in each semester were used to derive the offset and onset of LMS login activity. These parameters were determined using a threshold crossing method. The LMS activity threshold was calculated as 50% of the average normalized number of logins per bin (1 bin/288 bins × 0.5 = 0.001736 or 0.17%) (Supplementary Methods). Therefore, the LMS login offset was defined as the clock time when the normalized number of logins dropped and remained below the 50% threshold, and the LMS login onset was defined as the time point when logins exceeded and stayed above the 50% threshold (Extended Data Fig. 3). The LMS inactive period was defined as the duration of time from the LMS login offset to the LMS login onset. The midpoint of the LMS inactive period was also calculated between the LMS login offset and onset.

### Actigraphy study

NUS undergraduates aged 18–25 years were recruited to take part in a 6-week research study of their sleep–wake patterns during the school term. Participants were required to be non-smokers in good general health with a body mass index between 18.5 and 27.0 kg m^−^^2^. Individuals were ineligible if they reported shift work (paid work between 23:00 and 07:00) or if they planned on travelling across time zones during the study. Participants wore an actigraphy watch (Actiwatch Spectrum Plus or Actiwatch 2; Philips Respironics Inc.) on their non-dominant hand (Supplementary Methods) and made weekly visits to a classroom to have their data downloaded and to undergo a set of neurobehavioural tests (results not reported here). Among 202 undergraduate students who enroled in the study, 13 participants withdrew before the end of the data collection period (no longer available, *n* = 6; personal reasons, *n* = 5; falling ill, *n* = 2) and 5 participants were withdrawn from the study by the researchers for not complying with study procedures (for example, not wearing the actigraphy watch or not showing up on time for appointments). In the remaining 184 participants who wore the actigraphy watch for 6 weeks, two individuals were excluded because of poor quality data and one individual failed to provide his course timetable with his class start times. The dataset included 181 student participants with 7,329 nocturnal sleep recordings (range, 27–42 d per individual). The sample comprised students aged 21.3 ± 1.5 years (mean ± s.d.), including 115 women (63.5% female) and 162 Chinese (89.5%) enroled across four class years (number of students: first-year, *n* = 57; second-year, *n* = 59; third-year, *n* = 29; fourth-year, *n* = 35; missing data, *n* = 1). No statistical methods were used to predetermine the sample size for the actigraphy study but our sample size was similar to an earlier actigraphy study in high school students that compared sleep behaviour between different school start times^25^.

Actigraphy data were collected in 30-s epochs and analysed using Actiware software (v.6.0.9) (Supplementary Methods). The primary sleep variables were: (1) sleep onset, (2) sleep offset, (3) nocturnal total sleep time, (4) nocturnal time in bed for sleep and (5) midpoint of the sleep period. Each student’s actigraphy data were sorted by his/her first class of the day, and we restricted our analyses to class times in which there were at least 20 individuals whose first class of the day started at that time (08:00, *n* = 103; 09:00, *n* = 61; 10:00, *n* = 123; 11:00, *n* = 35; 12:00, *n* = 107; 14:00, *n* = 71; 16:00, *n* = 44). The cut-off of at least 20 students per class start time was chosen to ensure that we had enough participants to make meaningful comparisons between groups. The dataset comprised 3,701 nocturnal sleep recordings on school nights (at least one class occurred on the following day) and 3,129 nocturnal sleep recordings on non-school nights (no classes on the following day).

The frequency of instances in which students failed to wake up in time for class was evaluated by pooling data across participants for a given class start time. Two-sided chi-squared tests (SigmaPlot v.14.5, Systat Software, Inc.) were used to test for differences across class start times in the frequency of: (1) waking up after the start of class; and (2) waking up too late to reach class on time, which took into account travel time to reach school. The latter was assessed using the question ‘How long does it usually take for you to get from your residence to your first class of the day?’. The dataset comprised 6,546 sleep offset values from 174 participants who reported their travel time (start time of first class, number of sleep offset values: 08:00, 776 values; 09:00, 468 values; 10:00, 940 values; 11:00, 169 values; 12:00, 631 values; 14:00, 389 values; 16:00, 164 values; no class, 3,009 values).

The frequency of school days with naps was evaluated by pooling data across participants for different class start times. Naps were included in the analysis only if they were documented in a student’s daily diary and verified by their actigraphy record. A two-sided chi-squared test (SigmaPlot v.14.5, Systat Software, Inc.) was used to test for differences across class start times in the frequency of naps. The dataset comprised 336 school days with at least one nap (start time of first class, number of days with naps, number of days without naps: 08:00, 88 nap days, 716 non-nap days; 09:00, 50 nap days, 436 non-nap days; 10:00, 113 nap days, 889 non-nap days; 11:00, 18 nap days, 157 non-nap days; 12:00, 45 nap days, 633 non-nap days; 14:00, 14 nap days, 388 non-nap days; 16:00, 8 nap days, 162 non-nap days).

We tested whether population-derived LMS login parameters can reliably predict sleep behaviour in the average student by performing Pearson’s correlation analysis (two-sided test; SigmaPlot v.14.5, Systat Software, Inc.). Direct comparisons between individual observations were not possible because actigraphy-derived sleep parameters were measured in individual students (*n* = 181), whereas LMS-derived parameters were calculated for individual semesters (five consecutive semesters). We therefore computed average values for actigraphy parameters (averaged across participants) and LMS parameters (averaged across semesters) for each class start time (08:00, 09:00, 10:00, 11:00, 12:00, 14:00, 16:00 and non-school days) and entered these values into the correlation analysis (sleep onset versus LMS login offset; sleep offset versus LMS login onset; nocturnal total sleep time versus LMS inactive period; nocturnal time in bed for sleep versus LMS inactive period; midpoint of sleep versus midpoint of LMS inactive period). Pearson’s correlation analysis was also used to test for associations between sleep and LMS login behaviour on school days relative to non-school days (Δsleep onset versus ΔLMS login offset; Δsleep offset versus ΔLMS login onset; Δnocturnal total sleep time versus ΔLMS inactive period; Δnocturnal time in bed for sleep versus ΔLMS inactive period; Δmidpoint of sleep versus Δmidpoint of LMS inactive period). The best-fit linear regression line in each analysis was used to calculate the r.m.s.e. (the standard deviation of residuals), which was used to assess how well the LMS parameter predicted the corresponding sleep parameter.

### Academic performance

Students’ course grades were analysed over the six semesters for which Wi-Fi connection data and LMS data were available (2016/17 semester two, 2017/18 semester one, 2017/18 semester two, 2018/19 semester one, 2018/19 semester two, 2019/20 semester one). At NUS, students are given a letter grade that is converted to a number for calculating the grade point (A+ or A = 5.0, A− = 4.5, B+ = 4.0, B = 3.5, B− = 3.0, C+ = 2.5, C = 2.0, D+ = 1.5, D = 1.0, F = 0.0). Students earn course credits based on the estimated workload hours per week, and the grade point average represents the cumulative performance weighted by the credits earned in each course. Because a course can have multiple class start times (for example, 10:00 lecture on Monday and 16:00 tutorial on Wednesday), we decided to group data by morning and afternoon courses. Morning courses were defined as having all classes (for example, lectures, tutorials and laboratories) start before 12:00, and afternoon courses were defined as having all classes start at 12:00 or later. Mixed-timing courses were defined as having class meetings in the morning and afternoon. In each semester, we restricted our analyses to students who earned 20 course credits (the mode of the distribution for course credits) to ensure that they had a comparable total workload. This usually corresponded to taking four or five courses concurrently. The final sample included 33,818 unique students, ranging from 9,201 to 11,823 students per semester. Students’ demographic information is provided in Supplementary Table 1. The dataset comprised 275,192 individual course grades.

### Statistical models

The associations between class start time with Wi-Fi-confirmed attendance, LMS login parameters, actigraphy-derived sleep parameters and academic performance were examined using linear mixed-effects models (Supplementary Table 2). All models were implemented using the ‘lme4’ (v.1.1-29) and ‘lmerTest’ (v.3.1-3) packages with R statistical software (v.4.2)^61,62^. Models were fit by restricted maximum likelihood to estimate parameters associated with fixed and random effects. Model assumptions including linearity and normality of residuals were examined using the supplemental ‘redres’ package (v.0.0.0.9) to lme4. Satterthwaite’s method was used to perform two-tailed *t*-tests for fixed effects. In instances in which there was evidence of a statistically significant association between class start time and the outcome variable (*P* < 0.05), multiple comparisons between class start times were performed using Tukey’s test using the ‘emmeans’ package (v.1.6.1). Effect sizes were calculated with the ‘dabest’ package (v.0.3.0) using Python v.3.7.8 and R statistical software^63^.

The association between class start time (08:00, 09:00, 10:00, 12:00, 14:00, 16:00) and Wi-Fi-confirmed attendance was tested using a cross-classified model. Class start time was entered as a fixed effect factor (reference category = 08:00) with course module, school/faculty of enrolment and student included as crossed random effect factors. Covariates in the model included sex (male, female), age in years, ethnicity (Chinese, Indian, Malay, others), country of citizenship (Singaporean, Singapore permanent resident, foreigner), type of residence (on campus, off campus, mix of living on and off campus), students’ class year (Y1, Y2, Y3, Y4, Y5+) and semester (2018/19 semester one, 2018/19 semester two, 2019/20 semester one).

The association between class start time (08:00, 09:00, 10:00, 11:00, 12:00, 14:00, 16:00, no class) and each LMS-derived parameter (login offset, login onset, inactive period, midpoint of inactive period) was tested in separate linear mixed-effects models. Class start time was included as a fixed effect factor (reference category = no class) and semester was included as a random effect factor. Our analyses did not adjust for student-level characteristics (for example, age, sex, ethnicity or class year) or course-level characteristics because LMS login parameters were derived on a per-semester basis for each class start time using data that was pooled across students (that is, LMS login parameters were not determined in individual students or courses).

The association of students’ first class time of the day (08:00, 09:00, 10:00, 11:00, 12:00, 14:00, 16:00, no class) with each actigraphy-derived nocturnal sleep variable (sleep onset, sleep offset, total sleep time, time in bed for sleep, midpoint of sleep period) was tested in separate linear mixed-effects models. Class start time was entered in the model as a fixed effect factor (reference category = no class) with student included as a random effect factor. Covariates included age, sex, ethnicity, students’ class year and semester. Information on students’ citizenship, type of residence, course modules and school/faculty of enrolment were not included in the model because these data were not collected in the actigraphy study.

The association of grade point (individual course grade) with course start times (morning-only, afternoon-only, mix of morning and afternoon) was tested using a cross-classified model. Course start time was entered as a fixed factor (reference category = morning-only) with course module, student’s school/faculty of enrolment and student included as crossed random effect factors. Covariates included sex, age, ethnicity, citizenship, type of residence, students’ class year, semester and the proportion of morning classes that each student had during the semester. In a separate analysis, we tested the association between grade point average and the number of days per week that students had a morning class (0, 1, 2, 3, 4 or 5 d). The grade point average was calculated using all grades that a student obtained during a given semester, irrespective of the times that classes occurred. The cross-classified model included days per week with morning classes as a fixed effect factor (reference category = 0 d per week) with school/faculty of enrolment and student included as random effect factors. Covariates included sex, age, ethnicity, citizenship, type of residence, students’ class year and semester.

### Ethics statement

The research complied with all relevant ethical regulations. Permission to analyse university-archived data was obtained from the National University of Singapore (NUS) Institute for Applied Learning Sciences and Educational Technology (ALSET). ALSET stores and links de-identified student data for educational analytics research on the ALSET Data Lake. University-archived datasets included students’ demographic information (age, sex, ethnicity, year of matriculation, country of citizenship, type of residence), course enrolment, Wi-Fi connection data, use of the LMS and grades. Analyses of these data were approved by the NUS Learning and Analytics Committee on Ethics (LACE). University and course characteristics are described in the Supplementary Methods. Students whose university-archived data were included in our study provided informed consent to the NUS Student Data Protection Policy, which explains that their data can be used for research. Analyses of university-archived data were exempt from review by the NUS Institutional Review Board (IRB) because they were performed retrospectively on data that were de-identified to the researchers. Permission for collecting attendance data from course instructors was approved by LACE. Research procedures in the actigraphy study were approved by the NUS IRB and students provided written informed consent to take part in the research. Participants were paid $250 in Singapore dollars for completing the actigraphy study. The research analytical approach was not registered in advance.

### Reporting summary

Further information on research design is available in the Nature Portfolio Reporting Summary linked to this article.

## Supplementary information

Supplementary Information Supplementary Methods and Tables 1–5.

Reporting Summary

## Data Availability

The scored actigraphy data that supported the findings of this study are published with this article as Source data. University-archived data cannot be shared publicly because of legal and university restrictions where the research was conducted. In compliance with the Singapore Personal Data Protection Act, data stored on the National University of Singapore (NUS) Institute for Applied Learning Sciences and Educational Technology (ALSET) Data Lake are defined as personal data and cannot be shared publicly without student consent. Data can be accessed and analysed on the ALSET Data Lake server with approval by the NUS Learning and Analytics Committee on Ethics in accordance with NUS data management policies. Researchers who wish to access the data should contact ALSET at NUS (email: alsbox1@nus.edu.sg). Source data are provided with this paper.

## Source data

Source Data Fig. 2 Statistical source data.
Source Data Fig. 4 Statistical source data.

## Code availability

Custom code used to analyse university-archived data is stored with the data on the ALSET Data Lake server and can be accessed by contacting ALSET at NUS (email: alsbox1@nus.edu.sg).
