# Supplementary material for: Early morning university classes are associated with impaired sleep and academic performance
Source: Nat Hum Behav. 2023 Feb 20;7(4):502–14. doi: 10.1038/s41562-023-01531-x (PMC10129866; doi:10.1038/s41562-023-01531-x)
Supplement: Supplementary file 1 — Supplementary Methods and Tables 1–5. [file 41562_2023_1531_MOESM1_ESM.pdf]

nature human behaviour

Article
https://doi.org/10.1038/s41562-023-01531-x

Early morning university classes are associated with impaired sleep and academic performance

In the format provided by the authors and unedited

Supplementary Information

Supplementary Methods

University and course characteristics

The National University of Singapore (NUS) is a large public university with close to 30,000 undergraduate students enrolled per academic year. Our study included students who joined one of the following faculties or schools upon enrolling at the university (ranked from largest to smallest in size): Faculty of Arts & Social Sciences, Faculty of Engineering, Faculty of Science, Business School, School of Design and Environment, School of Computing, Faculty of Law, Yale-NUS College, Yong Loo Lin School of Medicine Nursing Program, School of Continuing and Lifelong Education, Yong Siew Toh Conservatory of Music, Multi-disciplinary Degree Programs. Most undergraduate degree programs span 4 academic years with two semesters per academic year (starting in August and January, respectively). Students took a combination of required and elective courses as part of their degree program. Students from the same school/faculty frequently enrolled in different courses (students choose their elective courses), and students who were enrolled in a given course could belong to different schools/faculties.

Most classes at NUS started on the hour between 08:00 and 18:00 (95% of all classes). We excluded data for the small proportion of classes that started at other times (e.g., 15 min, 30 min, or 45 min after the hour). The distribution of class start times varied substantially by time of day with nearly 80% of classes occurring on even hours (Percentage of classes by different start times: 08:00, 7.2%; 09:00, 7.5%; 10:00, 16.6%; 11:00, 4.1%; 12:00, 17.0%; 13:00, 4.3%; 14:00, 19.0%; 15:00, 3.8%; 16:00, 13.5%; 17:00, 1.6%; 18:00, 4.8%). The proportion of students taking classes at each start time varied by students' class year (Two-sided chi-squared test: $\chi^2(24)=7628$, Cramer's V = 0.042, 95% CI = 0.041 to 0.043, $P<0.001$) and by school/faculty (Two-sided chi-squared test: $\chi^2(72)=115152.8$, Cramer's V = 0.131, 95% CI = 0.130 to 0.132,

$P<0.001$). The number of students taking early morning classes was disproportionately higher in Year 1 and for students enrolled in the Faculty of Science or the Faculty of Engineering. Students' class year and school/faculty of enrolment were therefore included in statistical analyses of class start times in addition to other demographic variables.

Learning Management System (LMS) data analysis choices

We considered using different types of LMS actions (e.g., logins, downloads, uploads, logouts) to assess students' diurnal behaviour. We chose to analyse LMS logins because these were discrete events that required students to perform a clearly defined action (i.e., providing logon credentials to access the LMS). By comparison, the events logged during a given LMS interaction comprised a combination of student actions and automatic (computer-generated) actions that were not always clearly separable in the data logs. We did not consider logouts as a reliable indicator of behaviour because students could remain logged in while not interacting with the LMS, after which inactive users were eventually automatically logged out. Another reason for analysing LMS logins is that prior research demonstrated that these data can be used to perform profiling of students' diurnal learning-directed behaviour[1].

In exploratory analyses using data from one semester, students' logins were summed for different bin sizes (60 min, 15 min, 5 min, and 1 min) with the purpose of choosing the smallest bin size that resulted in a smooth diurnal distribution of logins for all class start times. Based on the empirical analysis, we chose to use 5-min bins for analysing LMS login data in each semester. For a given semester and class start time, the total number of logins per 5-min bin was summed across all students starting from 19:00 on the previous evening until 19:00 in the evening of the day in which the class took place (288 epochs per day). We chose 19:00 as the start and end point of each 24-h cycle because this allowed us to examine how students' nocturnal sleep/LMS login behaviour was related to their school start time on the following day (i.e., the nocturnal sleep opportunity preceded the school day). The same 24-h time window was examined on non-school days.

The LMS login offset and onset were determined using a threshold crossing method. The LMS activity threshold was determined empirically using two criteria: (1) the period of low LMS activity (i.e., below threshold) should span several hours during the night when students would normally be sleeping, and (2) the onset of LMS activity (i.e., crossing above threshold) should occur before students' first class of the day when students would usually be awake. For each semester and class start time, the LMS login time series was normalized by dividing the number of logins in each 5-min bin by the total number of logins across all bins (i.e., the sum across all bins was set to a value of 1). In the first iteration, we tested an LMS activity threshold that was equivalent to the average normalized activity per bin (i.e., 1 bin / 288 bins=0.003472). We did not use this threshold in our analyses because the downward and upward threshold crossings of LMS login activity occurred earlier and later than expected for sleep behaviour in university students. In the second iteration, the LMS activity threshold was calculated as 50% of the average normalized number of logins per bin (1 bin / 288 bins × 0.5=0.001736). We chose this threshold for our analyses because the duration and timing of low LMS activity met our predefined criteria and resembled sleep behaviour in university students. We did not attempt to optimize the LMS activity threshold to match our actigraphy-derived sleep parameters, nor did we test other LMS activity thresholds.

Actigraphy data collection and scoring

Students were instructed to wear the actigraphy watch at all times except when taking part in activities that might damage the device (e.g., contact sports or swimming). Participants were told to press an event marker button on their watch when going to bed/ waking up and when putting on/ taking off the actigraphy watch. The compliance rate for using the event marker was 64.0% for bedtimes and 63.9% for wake-up times (4,695 and 4,682 days, respectively). Participants were also required to complete a daily diary of times that they slept or removed the actigraphy watch. The compliance rate for completing diary entries was 96.5% for bedtimes and 96.8% for wake-up times (7,073 and 7,090 days, respectively). Event marker presses and diary

entries were missing for 3.4% of bedtimes and 3.1% of wake-up times (247 and 230 days, respectively).

Actigraphy data were collected in 30-s epochs and analysed using Actiware software (version 6.0.9). Time-in-bed intervals were marked in the actogram using participants' event marker presses and/or sleep diary entries. In participants with complete data, Pearson's correlation analysis (two-sided tests) showed that the times of event marker presses and diary entries were highly correlated for bedtime (Pearson's $r(4684)=0.981$, 95% CI = 0.980 to 0.982, $P<0.001$) and wake-up time (Pearson's $r(4671)=0.980$, 95% CI = 0.979 to 0.981, $P<0.001$). Event markers were prioritized over diary entries in instances where there was poor agreement between measures, while also taking into account each individual's pattern of activity and light exposure. The temporal discrepancy between event marker presses and diary entries differed by more than 30 min for 3.6% of bedtimes and 7.2% of wake-up times, and event markers were chosen in nearly all instances over diary entries (99.8% and 96.4% for bedtime and wake-up time, respectively). Among the 7,329 pairs of bedtimes and wake-up times in the dataset, 63.9% and 61.6% were scored using event marker presses, 27.4% and 30.9% were scored using diary entries, and 8.7% and 7.5% were based on participants' light/activity data in the actogram. Actograms were inspected, reviewed, and approved by all members of the research team before analysing the data to derive sleep variables. Sleep scoring of each time-in-bed interval was performed using the medium wake-sensitivity threshold (threshold=40 activity counts) and a 10-min immobility threshold for determining sleep onset and sleep offset.

References

1 Smarr, B. L. & Schirmer, A. E. 3.4 million real-world learning management system logins reveal the majority of students experience social jet lag correlated with decreased performance. *Scientific Reports* **8**, 4793, doi:10.1038/s41598-018-23044-8 (2018).

Supplementary Tables

Supplementary Table 1. Demographic characteristics of students included in different analyses.

	2016/17 semester 2	2017/18 semester 1	2017/18 semester 2	2018/19 semester 1	2018/19 semester 2	2019/20 semester 1
**Wi-Fi connection data (lectures with ≥100 students enrolled)**						
n	-	-	-	17,775	17,124	19,548
Age (mean ± SD)	-	-	-	21.0 ± 1.9	21.4 ± 1.8	21.3 ± 1.9
Female, *n* (%)	-	-	-	8,966 (50.4%)	8,683 (50.7%)	10,329 (52.8%)
Chinese, *n* (%)	-	-	-	15,457 (87.0%)	14,906 (87.0%)	16,965 (86.8%)
Class year, *n* (%)						
1	-	-	-	6,229 (35.0%)	6,242 (36.5%)	4,790 (24.5%)
2	-	-	-	5,389 (30.3%)	5,138 (30.0%)	6,219 (31.8%)
3	-	-	-	3,497 (19.7%)	3,345 (19.5%)	4,375 (22.4%)
4	-	-	-	2,402 (13.5%)	2,284 (13.3%)	3,732 (19.1%)
5+	-	-	-	258 (1.5%)	115 (0.7%)	432 (2.2%)
**Learning Management System data**						
n	22,580	24,615	22,996	25,506	24,121	-
Age (mean ± SD)	21.8 ± 2.3	21.5 ± 2.5	21.9 ± 2.4	21.5 ± 2.6	21.9 ± 2.5	-
Female, *n* (%)	11,829 (52.4%)	12,586 (51.1%)	11,823 (51.4%)	12,979 (50.9%)	12,343 (51.2%)	-
Chinese, *n* (%)	19,661 (87.1%)	21,305 (86.6%)	19,934 (86.7%)	22,019 (86.3%)	20,840 (86.4%)	-
Class year, *n* (%)						
1	6,417 (28.4%)	67,41 (27.4%)	6,577 (28.6%)	7,404 (29.0%)	7,365 (30.5%)	-
2	6,122 (27.1%)	6,420 (26.1%)	5,980 (26.0%)	6,625 (26.0%)	6,268 (26.0%)	-
3	5,027 (22.3%)	5,170 (21.0%)	4,984 (21.7%)	5,154 (20.2%)	4,913 (20.4%)	-
4	4,611 (20.4%)	5,532 (22.5%)	5,073 (22.1%)	5,508 (21.6%)	5,198 (21.5%)	-
5+	403 (1.8%)	752 (3.1%)	382 (1.7%)	815 (3.2%)	377 (1.6%)	-
**Grades data (students with 20 course credits)**						
n	10,110	10,643	9,269	11,423	9,201	11,823
Age (mean ± SD)	21.3 ± 1.8	20.9 ± 1.8	21.4 ± 1.7	20.8 ± 1.8	21.3 ± 1.8	20.7 ± 1.7
Female, *n* (%)	5,918 (58.5%)	5,851 (55.0%)	5,179 (55.9%)	6,300 (55.2%)	5,003 (54.4%)	6,117 (51.7%)
Chinese, *n* (%)	8,807 (87.1%)	9,343 (87.8%)	8,054 (86.9%)	9,929 (86.9%)	7,924 (86.1%)	10,081 (85.3%)
Class year, *n* (%)						
1	3,125 (30.9%)	4,163 (39.1%)	2,566 (27.7%)	4,964 (43.5%)	2,992 (32.5%)	5,252 (44.4%)
2	3,793 (37.5%)	2,954 (27.8%)	3,524 (38.0%)	3,234 (28.3%)	3,451 (37.5%)	3,647 (30.8%)
3	1,964 (19.4%)	2,241 (21.1%)	1,768 (19.1%)	2,056 (18.0%)	1,544 (16.8%)	1,830 (15.5%)
4	1,170 (11.6%)	1,132 (10.6%)	1,360 (14.7%)	1,025 (9.0%)	1,152 (12.5%)	891 (7.5%)
5+	58 (0.6%)	153 (1.4%)	51 (0.6%)	144 (1.3%)	62 (0.7%)	203 (1.7%)

Supplementary Table 2. Statistical models and parameter estimates

Linear mixed-effects models were fit by restricted maximum likelihood. Two-tailed t-tests used Satterthwaite's method. *P* values correspond to pairwise comparisons with the reference category before performing multiple comparison tests. Models were implemented using R statistical software.

Supp. Table 2a. Wi-Fi confirmed lecture attendance (%)

attendance ~ start_time + sex + age + ethnicity + citizenship + residence_type + class_year + semester + 1|course + 1|faculty + 1|student

Fixed effects

	Estimate	SE	*t*(df)	*P*
**Intercept**	59.53	4.43	13.5(369)	<0.001
**Class start time**				
08:00	ref	-	-	-
09:00	7.56	4.89	1.55(330)	0.123
10:00	11.10	3.70	3.00(328)	0.003
12:00	11.02	3.80	2.90(327)	0.004
14:00	10.82	3.77	2.87(328)	0.004
16:00	11.34	3.78	3.00(328)	0.003
**Sex**				
Female	ref	-	-	-
Male	-1.36	0.42	-3.24(22820)	0.001
**Age**	-2.21	0.14	-1.60(24430)	0.109
**Ethnicity**				
Chinese	ref	-	-	-
Indian	-6.65	0.70	-9.48(22110)	<0.001
Malay	-3.80	0.89	-4.28(21910)	<0.001
Others	-5.13	0.73	-7.06(22620)	<0.001
**Citizenship**				
Singapore	ref	-	-	-
Singapore PR	0.22	0.74	0.30(22570)	0.761
Foreigner	3.49	0.62	5.59(22980)	<0.001
**Residence type**				
Off campus	ref	-	-	-
On campus	-3.31	0.41	-8.71(21310)	<0.001
Mix	-3.87	0.38	-10.09(21810)	<0.001
**Class year**				
Year 1	ref	-	-	-
Year 2	-6.34	0.37	-17.29(58560)	<0.001
Year 3	-11.23	0.52	-21.51(41240)	<0.001
Year 4	-14.29	0.66	-21.79(33140)	<0.001
Year 5+	-20.05	1.33	-15.36(39230)	<0.001
**Semester**				
2018/19 sem 1	ref	-	-	-
2018/19 sem 2	-3.49	2.12	-1.65(327)	0.100
2019/20 sem 1	-2.42	1.98	-1.22(333)	0.223

Random effects (intercept): student, SD = 17.59; course module, SD = 15.05; school/faculty, SD = 4.00; residual, SD = 21.39

Supp. Table 2b. LMS login offset (h)

LMS_offset ~ start_time + 1|semester

Fixed effects

	Estimate	SE	*t*(df)	*P*
**Intercept**	25.85*	0.037	698(23)	<0.001
**Class start time**				
No class	ref	-	-	-
08:00	-0.43	0.046	-9.47(28)	<0.001
09:00	-0.32	0.046	-6.92(28)	<0.001
10:00	-0.22	0.046	-4.74(28)	<0.001
11:00	-0.25	0.046	-5.46(28)	<0.001
12:00	-0.18	0.046	-4.01(28)	<0.001
14:00	-0.28	0.046	-6.19(28)	<0.001
16:00	-0.28	0.046	-6.19(28)	<0.001

Random effects (intercept): semester, SD = 0.040; residual, SD = 0.072
*LMS login offset values were converted to decimal time for the analysis (25.85 = 01:51)

Supp. Table 2c. LMS login onset (h)

LMS_onset ~ start_time + 1|semester

Fixed effects

	Estimate	SE	*t*(df)	*P*
**Intercept**	32.68*	0.038	850(31)	<0.001
**Class start time**				
No class	ref	-	-	-
08:00	-1.37	0.052	-26.24(28)	<0.001
09:00	-0.87	0.052	-16.64(28)	<0.001
10:00	-0.58	0.052	-11.20(28)	<0.001
11:00	-0.17	0.052	-3.20(28)	0.003
12:00	-0.10	0.052	-1.92(28)	0.065
14:00	0.05	0.052	0.96(28)	0.345
16:00	0.12	0.052	2.24(28)	0.033

Random effects (intercept): semester, SD = 0.024; residual, SD = 0.082
*LMS login onset values were converted to decimal time for the analysis (32.68 = 08:41)

Supp. Table 2d. LMS inactive period (h)

LMS_inactive ~ start_time + 1|semester

Fixed effects

	**Estimate**	**SE**	***t*(df)**	***P***
**Intercept**	6.83	0.047	146.69(32)	<0.001
**Class start time**				
No class	ref	-	-	-
08:00	-0.93	0.066	-14.17(32)	<0.001
09:00	-0.55	0.066	-8.35(32)	<0.001
10:00	-0.37	0.066	-5.57(32)	<0.001
11:00	0.08	0.066	1.27(32)	0.215
12:00	0.08	0.066	1.27(32)	0.215
14:00	0.33	0.066	5.06(32)	<0.001
16:00	0.40	0.066	6.07(32)	<0.001

Random effects (intercept): semester, SD < 0.001; residual, SD = 0.10

Supp. Table 2e. LMS midpoint of inactive period (h)

LMS_mdpt ~ start_time + 1|semester

Fixed effects

	**Estimate**	**SE**	***t*(df)**	***P***
**Intercept**	29.27*	0.030	985.98(21)	<0.001
**Class start time**				
No class	ref	-	-	-
08:00	-0.90	0.036	-25.17(28)	<0.001
09:00	-0.59	0.036	-16.55(28)	<0.001
10:00	-0.40	0.036	-11.19(28)	<0.001
11:00	-0.21	0.036	-5.83(28)	<0.001
12:00	-0.14	0.036	-3.96(28)	<0.001
14:00	-0.12	0.036	-3.26(28)	0.003
16:00	-0.08	0.036	-2.33(28)	0.027

Random effects (intercept): semester, SD = 0.035; residual, SD = 0.057
*LMS midpoint values were converted to decimal time for the analysis (29.27 = 05:16)

Supp. Table 2f. Actigraphy-derived sleep onset (h)

sleep_onset ~ start_time + sex + age + ethnicity + class_year + semester + 1|student

Fixed effects

	Estimate	SE	*t*(df)	*P*
**Intercept**	25.07*	2.21	11.34(168)	<0.001
**Class start time**				
No class	ref	-	-	-
08:00	-0.38	0.054	-7.07(6585)	<0.001
09:00	-0.22	0.068	-3.26(6604)	0.001
10:00	-0.12	0.049	-2.51(6573)	0.012
11:00	-0.18	0.103	-1.71(6570)	0.087
12:00	-0.14	0.057	-2.44(6573)	0.015
14:00	-0.07	0.071	-1.03(6568)	0.303
16:00	-0.23	0.103	-2.24(6560)	0.025
**Sex**				
Female	ref	-	-	-
Male	-0.41	0.24	-1.70(168)	0.091
**Age**	0.06	0.10	0.59(168)	0.558
**Ethnicity**				
Chinese	ref	-	-	-
Indian	-0.52	1.18	-0.44(166)	0.662
Malay	-0.29	0.44	-0.66(167)	0.514
Others	0.11	0.39	0.27(168)	0.785
**Class year**				
Year 1	ref	-	-	-
Year 2	-0.04	0.25	-0.16(168)	0.873
Year 3	-0.54	0.33	-1.62(167)	0.107
Year 4	-0.25	0.38	-0.66(168)	0.513
**Semester**				
2018/19 sem 2	ref	-	-	-
2019/20 sem 1	0.20	0.19	1.06(168)	0.292

Random effects (intercept): student, SD = 1.14; residual, SD = 1.25
*Sleep onset values were converted to decimal time for the analysis (25.07 = 01:04)

Supp. Table 2g. Actigraphy-derived sleep offset (h)

sleep_offset ~ start_time + sex + age + ethnicity + class_year + semester + 1|student

Fixed effects

	Estimate	SE	*t*(df)	*P*
**Intercept**	32.49*	1.92	16.94(167)	<0.001
**Class start time**				
No class	ref	-	-	-
08:00	-1.66	0.060	-27.79(6616)	<0.001
09:00	-1.05	0.075	-13.92(6641)	<0.001
10:00	-0.83	0.054	-15.50(6600)	<0.001
11:00	-0.59	0.113	-5.18(6595)	<0.001
12:00	-0.40	0.062	-6.41(6599)	<0.001
14:00	-0.23	0.078	-2.90(6592)	0.004
16:00	-0.10	0.114	-0.85(6581)	0.397
**Sex**				
Female	ref	-	-	-
Male	-0.23	0.21	-1.11(167)	0.268
**Age**	0.04	0.09	0.46(167)	0.649
**Ethnicity**				
Chinese	ref	-	-	-
Indian	-0.27	1.02	-0.27(165)	0.791
Malay	-0.80	0.38	-2.10(167)	0.037
Others	-0.05	0.34	-0.14(167)	0.893
**Class year**				
Year 1	ref	-	-	-
Year 2	-0.45	0.21	-2.09(168)	0.038
Year 3	-0.38	0.29	-1.30(167)	0.194
Year 4	-0.49	0.33	-1.48(167)	0.141
**Semester**				
2018/19 sem 2	ref	-	-	-
2019/20 sem 1	0.38	0.16	2.31(167)	0.022

Random effects (intercept): student, SD = 0.98; residual, SD = 1.37
*Sleep offset values were converted to decimal time for the analysis (32.49 = 08:29)

Supp. Table 2h. Actigraphy-derived nocturnal total sleep time (h)

sleep_TST ~ start_time + sex + age + ethnicity + class_year + semester + 1|student

Fixed effects

	Estimate	SE	*t*(df)	*P*
**Intercept**	7.37	1.35	5.46(169)	<0.001
**Class start time**				
No class	ref	-	-	-
08:00	-1.16	0.059	-19.75(6698)	<0.001
09:00	-0.78	0.074	-10.69(6714)	<0.001
10:00	-0.65	0.053	-12.21(6678)	<0.001
11:00	-0.40	0.112	-3.53(6673)	<0.001
12:00	-0.23	0.061	-3.67(6677)	<0.001
14:00	-0.12	0.077	-1.58(6667)	0.113
16:00	0.11	0.112	0.97(6652)	0.334
**Sex**				
Female	ref	-	-	-
Male	0.25	0.15	1.70(169)	0.091
**Age**	-0.05	0.06	-0.85(169)	0.395
**Ethnicity**				
Chinese	ref	-	-	-
Indian	0.03	0.72	0.04(165)	0.966
Malay	-0.45	0.27	-1.70(168)	0.090
Others	-0.17	0.24	-0.71(168)	0.479
**Class year**				
Year 1	ref	-	-	-
Year 2	-0.33	0.15	-2.18(170)	0.031
Year 3	0.14	0.20	0.70(168)	0.488
Year 4	-0.10	0.23	-0.43(169)	0.665
**Semester**				
2018/19 sem 2	ref	-	-	-
2019/20 sem 1	0.13	0.12	1.12(169)	0.267

Random effects (intercept): student, SD = 0.67; residual, SD = 1.36

Supp. Table 2i. Actigraphy-derived nocturnal time in bed for sleep (h)

sleep_TIB ~ start_time + sex + age + ethnicity + class_year + semester + 1|student

Fixed effects

	Estimate	SE	*t*(df)	*P*
**Intercept**	7.91	1.62	4.89(169)	<0.001
**Class start time**				
No class	ref	-	-	-
08:00	-1.32	0.065	-20.17(6688)	<0.001
09:00	-0.84	0.082	-10.23(6709)	<0.001
10:00	-0.71	0.059	-12.07(6667)	<0.001
11:00	-0.32	0.124	-2.58(6661)	0.010
12:00	-0.23	0.068	-3.44(6665)	<0.001
14:00	-0.09	0.085	-1.02(6656)	0.308
16:00	0.16	0.125	1.30(6641)	0.194
**Sex**				
Female	ref	-	-	-
Male	0.04	0.18	0.22(169)	0.830
**Age**	-0.01	0.08	-0.17(169)	0.868
**Ethnicity**				
Chinese	ref	-	-	-
Indian	0.55	0.86	0.64(165)	0.521
Malay	-0.63	0.32	-1.99(168)	0.049
Others	-0.04	0.29	-0.01(168)	0.990
**Class year**				
Year 1	ref	-	-	-
Year 2	-0.51	0.18	-2.79(169)	0.006
Year 3	0.18	0.24	0.72(168)	0.473
Year 4	-0.34	0.28	-1.22(168)	0.226
**Semester**				
2018/19 sem 2	ref	-	-	-
2019/20 sem 1	0.25	0.14	1.82(169)	0.070

Random effects (intercept): student, SD = 0.81; residual, SD = 1.51

Supp. Table 2j. Actigraphy-derived midpoint of sleep (h)

sleep_mdpt ~ start_time + sex + age + ethnicity + class_year + semester + 1|student

Fixed effects

	Estimate	SE	*t*(df)	*P*
**Intercept**	28.77*	1.95	14.79(168)	<0.001
**Class start time**				
No class	ref	-	-	-
08:00	-1.02	0.047	-21.78(6583)	<0.001
09:00	-0.64	0.059	-10.76(6602)	<0.001
10:00	-0.48	0.042	-11.36(6572)	<0.001
11:00	-0.39	0.089	-4.34(6568)	<0.001
12:00	-0.27	0.049	-5.52(6571)	<0.001
14:00	-0.15	0.061	-2.43(6566)	0.015
16:00	-0.17	0.089	-1.85(6559)	0.064
**Sex**				
Female	ref	-	-	-
Male	-0.32	0.21	-1.51(168)	0.132
**Age**	0.05	0.09	0.56(168)	0.574
**Ethnicity**				
Chinese	ref	-	-	-
Indian	-0.39	1.04	-0.38(166)	0.705
Malay	-0.54	0.38	-1.41(167)	0.161
Others	0.03	0.35	0.09(167)	0.925
**Class year**				
Year 1	ref	-	-	-
Year 2	-0.24	0.22	-1.12(168)	0.263
Year 3	-0.46	0.29	-1.56(167)	0.120
Year 4	-0.37	0.33	-1.10(168)	0.273
**Semester**				
2018/19 sem 2	ref	-	-	-
2019/20 sem 1	0.29	0.17	1.74(168)	0.084

Random effects (intercept): student, SD = 1.01; residual, SD = 1.08
*Sleep midpoint values were converted to decimal time for the analysis (28.77 = 04:46)

Supp. Table 2k. Course grades (grade point)

grade_point ~ course_start_time + proportion_AM + sex + age + ethnicity + citizenship + residence_type + class_year + semester + 1|course + 1|faculty + 1|student

Fixed effects

	Estimate	SE	*t*(df)	*P*
**Intercept**	5.016	0.087	57.41(48)	<0.001
**Course start time**				
Morning-only	ref	-	-	-
Afternoon-only	0.016	0.005	3.18(79290)	0.001
Mixed morning/afternoon	-0.001	0.005	-0.22(105200)	0.823
**% morning classes**	0.003	0.012	0.26(259800)	0.796
**Sex**				
Female	ref	-	-	-
Male	0.231	0.009	25.62(35060)	<0.001
**Age**	-0.064	0.003	-20.35(38000)	<0.001
**Ethnicity**				
Chinese	ref	-	-	-
Indian	-0.167	0.015	-11.03(33240)	<0.001
Malay	-0.272	0.018	-14.73(32580)	<0.001
Others	-0.169	0.015	-11.48(33060)	<0.001
**Citizenship**				
Singapore	ref	-	-	-
Singapore PR	0.023	0.015	1.48(33690)	0.138
Foreigner	0.019	0.013	1.47(34270)	0.140
**Residence type**				
Off campus	ref	-	-	-
On campus	-0.008	0.009	-0.81(34710)	0.421
Mix	-0.047	0.008	-5.81(32360)	<0.001
**Class year**				
Year 1	ref	-	-	-
Year 2	0.111	0.006	17.72(123900)	<0.001
Year 3	0.179	0.009	19.09(71090)	<0.001
Year 4	0.278	0.013	21.32(56560)	<0.001
Year 5+	0.257	0.025	10.30(95910)	<0.001
**Semester**				
2016/17 sem 2	ref	-	-	-
2017/18 sem 1	-0.069	0.012	-5.65(7548)	<0.001
2017/18 sem 2	-0.042	0.012	-3.46(7857)	<0.001
2018/19 sem 1	-0.119	0.014	-8.74(11290)	<0.001
2018/19 sem 2	-0.083	0.014	-6.12(12190)	<0.001
2019/20 sem 1	-0.133	0.016	-8.46(18000)	<0.001

Random effects (intercept): student, SD = 0.524; course module, SD = 0.222; school/faculty, SD = 0.212; residual, SD = 0.655

Supp. Table 2I. Grade point average (GPA)

grade_point_average ~ days_AM_class + sex + age + ethnicity + citizenship + residence_type + class_year + semester + 1|faculty + 1|student

Fixed effects

	Estimate	SE	*t*(df)	*P*
**Intercept**	4.874	0.076	64.40(79)	<0.001
**Days per week with morning classes**				
0	ref	-	-	-
1	-0.069	0.013	-5.43(50160)	<0.001
2	-0.103	0.012	-8.43(50340)	<0.001
3	-0.117	0.012	-9.53(50410)	<0.001
4	-0.141	0.013	-11.24(50180)	<0.001
5	-0.146	0.014	-10.74(50060)	<0.001
**Sex**				
Female	ref	-	-	-
Male	0.186	0.009	21.80(34230)	<0.001
**Age**	-0.050	0.003	-17.17(35940)	<0.001
**Ethnicity**				
Chinese	ref	-	-	-
Indian	-0.160	0.015	-10.97(33840)	<0.001
Malay	-0.247	0.018	-13.97(32350)	<0.001
Others	-0.155	0.014	-11.00(33200)	<0.001
**Citizenship**				
Singapore	ref	-	-	-
Singapore PR	0.016	0.015	1.10(34160)	0.272
Foreigner	0.013	0.012	1.06(34500)	0.288
**Residence type**				
Off campus	ref	-	-	-
On campus	-0.0007	0.009	-0.08(35070)	0.937
Mix	-0.048	0.008	-6.19(32090)	<0.001
**Class year**				
Year 1	ref	-	-	-
Year 2	0.078	0.005	14.55(61980)	<0.001
Year 3	0.163	0.008	19.56(54760)	<0.001
Year 4	0.341	0.012	29.03(48270)	<0.001
Year 5+	0.324	0.023	13.80(61840)	<0.001
**Semester**				
2016/17 sem 2	ref	-	-	-
2017/18 sem 1	-0.044	0.007	-6.37(56350)	<0.001
2017/18 sem 2	-0.037	0.007	-5.25(55080)	<0.001
2018/19 sem 1	-0.091	0.009	-10.31(58680)	<0.001
2018/19 sem 2	-0.084	0.009	-9.23(60020)	<0.001
2019/20 sem 1	-0.115	0.011	-10.05(46930)	<0.001

Random effects (intercept): student, SD = 0.483; school/faculty, SD = 0.161; residual, SD = 0.364

Supplementary Table 3. Learning Management System (LMS)-derived parameters sorted by students' first class of the day.

		Start time of first class (hh:mm)							
**LMS parameter**	**Semester**	**08:00**	**09:00**	**10:00**	**11:00**	**12:00**	**14:00**	**16:00**	**No class**
LMS login offset (hh:mm)	2016/17 Sem 2	01:25	01:30	01:35	01:30	01:45	01:30	01:30	01:50
	2017/18 Sem 1	01:25	01:30	01:35	01:35	01:40	01:40	01:25	01:45
	2017/18 Sem 2	01:25	01:35	01:45	01:45	01:50	01:35	01:40	01:55
	2018/19 Sem 1	01:25	01:30	01:35	01:35	01:35	01:30	01:40	01:55
	2018/19 Sem 2	01:25	01:35	01:40	01:35	01:30	01:35	01:35	01:50
LMS login onset (hh:mm)	2016/17 Sem 2	07:30	07:50	08:05	08:25	08:30	08:35	08:50	08:40
	2017/18 Sem 1	07:20	07:45	08:05	08:25	08:35	08:40	08:45	08:40
	2017/18 Sem 2	07:20	07:55	08:05	08:40	08:40	08:45	08:50	08:40
	2018/19 Sem 1	07:10	07:45	08:05	08:30	08:35	08:50	08:40	08:45
	2018/19 Sem 2	07:15	07:50	08:10	08:35	08:35	08:50	08:55	08:40
Duration of LMS inactive period (h)	2016/17 Sem 2	6.1	6.3	6.5	6.9	6.8	7.1	7.3	6.8
	2017/18 Sem 1	5.9	6.2	6.5	6.8	6.9	7.0	7.3	6.9
	2017/18 Sem 2	5.9	6.3	6.3	6.9	6.8	7.2	7.2	6.7
	2018/19 Sem 1	5.7	6.2	6.5	6.9	7.0	7.3	7.0	6.8
	2018/19 Sem 2	5.8	6.3	6.5	7.0	7.1	7.3	7.3	6.8
Midpoint of LMS inactive period (hh:mm)	2016/17 Sem 2	04:28	04:40	04:50	04:57	05:08	05:03	05:10	05:15
	2017/18 Sem 1	04:22	04:38	04:50	05:00	05:08	05:10	05:05	05:12
	2017/18 Sem 2	04:22	04:45	04:55	05:12	05:15	05:10	05:15	05:17
	2018/19 Sem 1	04:17	04:38	04:50	05:02	05:05	05:10	05:10	05:20
	2018/19 Sem 2	04:20	04:43	04:55	05:05	05:03	05:13	05:15	05:15

Supplementary Table 4. Actigraphy-derived sleep parameters sorted by students' first class of the day. The mean ± SD is shown for each sleep variable. TST = total sleep time; TIB = time in bed.

Start time of first class (hh:mm)	*n*	Sleep onset (hh:mm)	Sleep offset (hh:mm)	Nocturnal TST (h)	Nocturnal TIB (h)	Midpoint of sleep (hh:mm)
08:00	103	01:35 ± 01:13	07:18 ± 01:18	5.2 ± 1.1	6.2 ± 1.3	04:28 ± 01:05
09:00	61	01:50 ± 01:14	07:56 ± 01:06	5.5 ± 1.0	6.7 ± 1.1	04:56 ± 01:06
10:00	123	01:58 ± 01:20	08:11 ± 00:58	5.6 ± 0.9	6.8 ± 1.0	05:06 ± 01:04
11:00	35	02:21 ± 01:26	08:37 ± 01:04	5.7 ± 1.3	6.9 ± 1.3	05:33 ± 01:04
12:00	107	01:48 ± 01:18	08:37 ± 01:13	6.2 ± 0.9	7.4 ± 1.1	05:13 ± 01:09
14:00	71	02:02 ± 01:36	08:49 ± 01:26	6.0 ± 1.2	7.4 ± 1.4	05:24 ± 01:21
16:00	44	01:33 ± 01:23	08:54 ± 01:21	6.4 ± 1.0	7.8 ± 1.3	05:12 ± 01:12
No class	181	01:59 ± 01:14	09:04 ± 01:18	6.3 ± 0.9	7.6 ± 1.0	05:31 ± 01:09

Supplementary Table 5. Effect sizes of different class start times for actigraphy-derived sleep parameters relative to days with no classes. Unstandardized and standardized effects sizes are shown without adjustment for covariates.

		Change relative to non-school days	
**Sleep variable**	**Start time of first class (hh:mm)**	**Mean difference in hours (95% CI)**	**Cohen's d (95% CI)**
Sleep onset	08:00	-0.38 (-0.55 to -0.22)	-0.30 (-0.44 to -0.17)
	09:00	-0.27 (-0.48 to -0.05)	-0.21 (-0.39 to -0.03)
	10:00	-0.11 (-0.27 to 0.05)	-0.08 (-0.21 to 0.04)
	11:00	-0.21 (-0.46 to 0.01)	-0.15 (-0.37 to 0.01)
	12:00	-0.16 (-0.30 to -0.02)	-0.13 (-0.24 to -0.01)
	14:00	0.05 (-0.16 to 0.26)	0.03 (-0.12 to 0.18)
	16:00	-0.29 (-0.50 to -0.06)	-0.21 (-0.40 to -0.04)
Sleep offset	08:00	-1.72 (-1.99 to -1.43)	-1.29 (-1.59 to -1.00)
	09:00	-1.12 (-1.44 to -0.81)	-0.90 (-1.23 to -0.53)
	10:00	-0.94 (-1.17 to -0.75)	-0.85 (-1.07 to -0.62)
	11:00	-0.97 (-1.37 to -0.60)	-0.75 (-1.08 to -0.46)
	12:00	-0.55 (-0.72 to -0.38)	-0.43 (-0.56 to -0.30)
	14:00	-0.20 (-0.42 to 0.00)	-0.14 (-0.31 to 0.01)
	16:00	-0.25 (-0.49 to -0.03)	-0.19 (-0.38 to -0.02)
Nocturnal time in bed for sleep	08:00	-1.35 (-1.57 to -1.12)	-1.14 (-1.39 to -0.89)
	09:00	-0.80 (-1.08 to -0.53)	-0.74 (-1.04 to -0.45)
	10:00	-0.83 (-1.02 to -0.64)	-0.80 (-1.00 to -0.60)
	11:00	-0.56 (-0.95 to -0.08)	-0.50 (-0.85 to -0.02)
	12:00	-0.43 (-0.64 to -0.24)	-0.42 (-0.61 to -0.22)
	14:00	-0.24 (-0.48 to 0.00)	-0.19 (-0.39 to 0.00)
	16:00	-0.16 (-0.45 to 0.11)	-0.14 (-0.39 to 0.11)
Nocturnal total sleep time	08:00	-1.16 (-1.37 to -0.95)	-1.13 (-1.37 to -0.89)
	09:00	-0.70 (-0.94 to -0.47)	-0.74 (-1.02 to -0.44)
	10:00	-0.70 (-0.88 to -0.53)	-0.74 (-0.93 to -0.55)
	11:00	-0.53 (-0.89 to -0.15)	-0.48 (-0.78 to -0.11)
	12:00	-0.31 (-0.49 to -0.13)	-0.34 (-0.54 to -0.14)
	14:00	-0.17 (-0.37 to 0.02)	-0.16 (-0.36 to 0.02)
	16:00	-0.12 (-0.37 to 0.13)	-0.13 (-0.39 to 0.14)
Midpoint of sleep	08:00	-1.02 (-1.21 to -0.83)	-0.89 (-1.12 to -0.67)
	09:00	-0.59 (-0.82 to -0.36)	-0.51 (-0.74 to -0.25)
	10:00	-0.47 (-0.62 to -0.33)	-0.43 (-0.60 to -0.27)
	11:00	-0.47 (-0.68 to -0.24)	-0.40 (-0.62 to -0.20)
	12:00	-0.35 (-0.46 to -0.23)	-0.30 (-0.40 to -0.20)
	14:00	-0.08 (-0.25 to 0.09)	-0.07 (-0.20 to 0.07)
	16:00	-0.28 (-0.44 to -0.10)	-0.23 (-0.39 to -0.08)
