# Supplementary material for: Early morning university classes are associated with impaired sleep and academic performance
Source: Nat Hum Behav. 2023 Feb 20;7(4):502–14. doi: 10.1038/s41562-023-01531-x (PMC10129866; doi:10.1038/s41562-023-01531-x)
Supplement: Supplementary file 2 — Reporting Summary [file 41562_2023_1531_MOESM2_ESM.pdf]

nature portfolio

Corresponding author(s): Joshua J GOOLEY

Last updated by author(s): Nov 1, 2022

Reporting Summary

Nature Portfolio wishes to improve the reproducibility of the work that we publish. This form provides structure for consistency and transparency in reporting. For further information on Nature Portfolio policies, see our Editorial Policies and the Editorial Policy Checklist.

Statistics

For all statistical analyses, confirm that the following items are present in the figure legend, table legend, main text, or Methods section.

n/a	Confirmed	
☐	☒	The exact sample size (*n*) for each experimental group/condition, given as a discrete number and unit of measurement
☐	☒	A statement on whether measurements were taken from distinct samples or whether the same sample was measured repeatedly
☐	☒	The statistical test(s) used AND whether they are one- or two-sided<br>*Only common tests should be described solely by name; describe more complex techniques in the Methods section.*
☐	☒	A description of all covariates tested
☐	☒	A description of any assumptions or corrections, such as tests of normality and adjustment for multiple comparisons
☐	☒	A full description of the statistical parameters including central tendency (e.g. means) or other basic estimates (e.g. regression coefficient) AND variation (e.g. standard deviation) or associated estimates of uncertainty (e.g. confidence intervals)
☐	☒	For null hypothesis testing, the test statistic (e.g. F, t, r) with confidence intervals, effect sizes, degrees of freedom and P value noted<br>*Give P values as exact values whenever suitable.*
☒	☐	For Bayesian analysis, information on the choice of priors and Markov chain Monte Carlo settings
☒	☐	For hierarchical and complex designs, identification of the appropriate level for tests and full reporting of outcomes
☐	☒	Estimates of effect sizes (e.g. Cohen's *d*, Pearson's *r*), indicating how they were calculated

Our web collection on statistics for biologists contains articles on many of the points above.

Software and code

Policy information about availability of computer code

Data collection	No software was used to collect data in this study.
Data analysis	In the actigraphy study, data were analyzed using Actiware software (version 6.0.9).<br><br>Pearson's correlation analysis and chi-squared tests were performed using SigmaPlot software (version 14.5, Systat Software, Inc.).<br><br>Linear mixed-effects models were implemented using the "lme4" (version 1.1-29) and "lmerTest" (version 3.1-3) packages in R Statistical Software (version 4.2). Model assumptions were examined using the supplemental "redres" package (version 0.0.0.9) to lme4. Multiple comparisons were performed using Tukey's test using the "emmeans" package (version 1.6.1). Effect sizes were calculated with the “dabest” package (version 0.3.0) using Python 3.7.8 and R statistical software.

For manuscripts utilizing custom algorithms or software that are central to the research but not yet described in published literature, software must be made available to editors and reviewers. We strongly encourage code deposition in a community repository (e.g. GitHub). See the Nature Portfolio guidelines for submitting code & software for further information.

Data

Policy information about availability of data

All manuscripts must include a data availability statement. This statement should provide the following information, where applicable:

- Accession codes, unique identifiers, or web links for publicly available datasets
- A description of any restrictions on data availability
- For clinical datasets or third party data, please ensure that the statement adheres to our policy

The actigraphy data that support the findings of this study are available as source data (Supplementary Information) with the published article. University-archived data cannot be shared publicly because of legal and university restrictions where the research was conducted. In compliance with the Singapore Personal Data Protection Act, data stored on the NUS Institute for Applied Learning Sciences and Educational Technology (ALSET) Data Lake is defined as personal data and cannot be shared publicly without student consent. Data can be accessed and analyzed on the ALSET Data Lake server with approval by the NUS Learning Analytics Committee on Ethics, in accordance with NUS data management policies. Researchers who wish to access the data should contact ALSET at NUS (email: alsbox1@nus.edu.sg).

Field-specific reporting

Please select the one below that is the best fit for your research. If you are not sure, read the appropriate sections before making your selection.

☐ Life sciences ☒ Behavioural & social sciences ☐ Ecological, evolutionary & environmental sciences

For a reference copy of the document with all sections, see nature.com/documents/nr-reporting-summary-flat.pdf

Behavioural & social sciences study design

All studies must disclose on these points even when the disclosure is negative.

Study description

The study included (1) retrospective analyses of university-archived student data and (2) analyses of university students' sleep behavior using actigraphy. All data are quantitative.

Research sample

The sample comprised undergraduate students enrolled at the National University of Singapore (NUS). University-archived student data were representative of students enrolled at NUS (average age of 21 years, 51% female, 87% Chinese) because we used all available data of the student population. Actigraphy studies were performed in a smaller sample of students who were recruited from the general student population. The sample was representative of students at NUS but included a higher percentage of women (average age of 21 years, 64% female, 89% Chinese). The rationale for studying these samples was to test the hypothesis that early morning classes result in lower class attendance, shorter sleep, and poorer grades.

Sampling strategy

Analyses of students' university-archived data included Wi-Fi connection logs, Learning Management System logins and grades. No sample size calculation was performed because we performed a retrospective analysis of all available student data.

Analyses of actigraphy data were based on a 6-week study of students' natural sleep behavior during the school semester. The data were collected to investigate relationships between students' sleep behavior and neurobehavioral performance (results not reported here). The sample of 181 students was sufficient for comparing sleep behavior between different class start times because each student contributed data for multiple class start times. We restricted our analyses to class start times in which there were at least 20 individuals whose first class of the day started at that time (08:00, n=103; 09:00, n=61, 10:00, n=123, 11:00, n=35, 12:00, n=107; 14:00, n=71; 16:00, n=44) to ensure that we had enough participants to make meaningful comparisons between groups. The dataset comprised 3,701 nocturnal sleep recordings on school nights and 3,129 nocturnal sleep recordings on non-school nights. Effect sizes of class start times for the primary sleep variables (wake-up time and nocturnal sleep duration) were medium-to-large. No statistical methods were used to pre-determine the sample size for the actigraphy study. However, the sample size was comparable to prior studies conducted in high school students that compared sleep behaviour between different school start times (e.g., Dunster et al., Sleepmore in Seattle: Later school start times are associated with more sleep and better performance in high school students. Science Advances 4, 2018).

Data collection

University-archived datasets were obtained from the National University of Singapore (NUS) Institute for Applied Learning Sciences and Educational Technology (ALSET). ALSET stores and links de-identified student data for educational analytics research. University-archived datasets included students' demographic information (age, sex, ethnicity, year of matriculation), course enrolment and class timetables, Wi-Fi connection data, Learning Management System (LMS) data, and grades. Demographic information, course enrolment and class timetables, and grades were provided by the NUS Registrar's Office which is responsible for keeping all student records. Wi-Fi connection data and LMS data were provided by NUS Information Technology (IT). The NUS wireless network comprises several thousand Wi-Fi access points. Each time that a student's Wi-Fi enabled device associated with the NUS wireless network the transmission data were logged. Students' Wi-Fi connection data were added to the ALSET Data Lake by a data pipeline managed by NUS IT. Each data point included the tokenized student identity, the anonymised media access control (MAC) address used to identify the Wi-Fi enabled device (e.g., smartphone, laptop, or tablet), the name and location descriptor of the Wi-Fi access point, and the start and end time of each Wi-Fi connection. LMS login data were extracted from students' logged interactions with the NUS Integrated Virtual Learning Environment (IVLE). The IVLE is a LMS designed and built by NUS for administering course content. Each data point included the type of student interaction (e.g., login, download, upload, logoff) and timestamp. NUS IT was responsible for merging all data with the ALSET Data Lake. The same student-specific tokens were represented across data tables, allowing for different types of data to be combined without knowing students' identities.

The researchers were not present during collection of university-archived datasets. The data were collected from naturally behaving students who were using the university's resources (e.g., Wi-Fi network and LMS) as part of normal student life. During the period of data collection the students would have interacted with other individuals on campus, including other students, faculty, full-time employees of the university, and visitors. The researchers only had access to student data on the ALSET Data Lake. The researchers were not blinded to the experimental conditions (i.e., students' class start time) or study hypothesis when analysing the data.

Actigraphy data were collected from NUS undergraduates who were recruited to take part in a 6-week research study of their sleep-wake patterns during the school semester. Participants wore an actigraphy watch (Actiwatch Spectrum Plus or Actiwatch 2; Philips Respironics Inc., Pittsburgh, PA) on their non-dominant hand and made weekly visits to a classroom to have their data downloaded by the researchers. Students submitted their class timetable at the end of the study period. The researchers were not blinded to the experimental conditions of the study (i.e., students' class start time) or the study hypothesis. The researchers checked whether participants complied with wearing the actigraphy watch, but they did not analyze the data until after all data was collected. Actograms were inspected, reviewed, and approved by all members of the research team before analyzing the data to derive sleep variables. Subsequently, the sleep data were sorted by students' first class of the day using their class timetable.

Timing

University-archived data were analyzed using all available data on the ALSET Data Lake prior to the COVID-19 pandemic: (1) Wi-Fi connection data were analyzed from August 2018 to December 2019 (3 semesters), (2) Learning Management System login data were analyzed from January 2017 to May 2019 (5 semesters), and (3) grades data were analyzed from January 2017 to December 2019 (6 semesters).

Actigraphy studies were performed during the following 6-week periods: (1) January 4, 2019 to February 15, 2019, (2) March 1, 2019 to April 12, 2019, (3) September 27, 2019 to Nov 8, 2019.

Data exclusions

In analyses of university-archived student data:
(1) Wi-confirmed attendance was investigated only for courses that (i) were categorized as a lecture course according to the university timetable, (ii) were held once per week, (iii), were held at least 7 times over the 13-week semester, (iv) lasted 2 h per session, and (v) had an enrollment of at least 100 undergraduate students. The rationale for these criteria was to ensure that comparable types of courses were included in analyses across different class start times. Among the 436 courses that met these criteria, 71 were excluded due to missing or incomplete Wi-Fi connection data or inconsistencies with the class timetable (e.g., due to cancelled or rescheduled classes). The remaining 365 courses were sorted by their start time, and data were analyzed only for those start times in which there were at least 5 courses per semester. This ensured that all class start times included at least 15 different courses spanning a comparable time period (08:00, 21 courses; 09:00, 18 courses; 10:00, 89 courses; 12:00, 67 courses; 14:00, 72 courses; 16:00, 70 courses). The final dataset included 337 courses and 23,391 unique students.
(2) All available Learning Management System (LMS) data were used. There were no exclusionary criteria.
(3) Grade point average was analyzed in students who earned 20 course credits in a given semester. The rationale for this criterion was to ensure that students in our analyses had a comparable total workload. The criterion was determined by taking the mode of the distribution for course credits.

In the actigraphy study, data were excluded (i) for 2 individuals because of poor quality data (the researchers could not determine the time-in-bed intervals for sleep scoring), and (ii) for 1 individual who failed to provide his course timetable with his class start times. The dataset included 181 student participants with 7,329 nocturnal sleep recordings (range, 27-42 days per individual).

Non-participation

In analyses of university-archived student data, the issue of non-participation is not applicable.

In the actigraphy study, there were 202 undergraduate students who enrolled in the study. There were 13 participants who withdrew before the end of the data collection period (no longer available, n = 6; personal reasons, n = 5; falling ill, n = 2), and 5 participants who were withdrawn from the study by the researchers for not complying with study procedures (e.g., not wearing the actigraphy watch or not showing up on time for appointments).

Randomization

Students were not allocated into experimental groups. Students’ timetables were used to sort their data (attendance, sleep, grades) by different class start times.

Reporting for specific materials, systems and methods

We require information from authors about some types of materials, experimental systems and methods used in many studies. Here, indicate whether each material, system or method listed is relevant to your study. If you are not sure if a list item applies to your research, read the appropriate section before selecting a response.

Materials & experimental systems

n/a	Involved in the study	
☒	☐	Antibodies
☒	☐	Eukaryotic cell lines
☒	☐	Palaeontology and archaeology
☒	☐	Animals and other organisms
☐	☒	Human research participants
☒	☐	Clinical data
☒	☐	Dual use research of concern

Methods

n/a	Involved in the study	
☒	☐	ChIP-seq
☒	☐	Flow cytometry
☒	☐	MRI-based neuroimaging

Human research participants

Policy information about studies involving human research participants

Population characteristics	See above
Recruitment	Analyses of university-archived student data did not involve participant recruitment.<br><br>The method of estimating Wi-Fi confirmed attendance requires that students have a Wi-Fi enabled device that is actively scanning for wireless access points. Some students may have disabled Wi-Fi scanning on their devices or used a cellular data plan instead. This may have introduced some sample bias; however, Wi-Fi confirmed attendance underestimated instructor-reported attendance by only a small amount and these variables were strongly correlated. Our analyses focused on large lecture courses (>100 enrolled students) because we expected that students would be more likely to skip these classes compared with other types of courses that are smaller and more interactive. In our dataset, large lecture courses were more likely to be taken by first-year students, and early class start times were more common for students enrolled in science and engineering programs. To address these potential sources of bias, our statistical models included students' class year and school/faculty of enrollment. Hence, we consider it unlikely that sample bias altered the study results substantially.<br><br>We used Learning Management System (LMS) interactions to estimate students' sleep opportunities. Students' interactions with the LMS may be influenced by factors unrelated to sleep-wake behaviour, e.g. personal preferences or social schedules. Additionally, the LMS may not be required for some types of courses. These factors may have contributed to sample bias. We consider it unlikely that this affected the results of the study because most students regularly used the LMS and the findings were reproduced in the actigraphy study.<br><br>Participants were recruited for the actigraphy study by placing advertisements on student digital platforms (e.g., the student portal for jobs and research studies), posting flyers on campus, and in-person recruitment booths at approved locations on campus. Students who expressed interest in the study were given a one-page description of the research study. Individuals who remained interested were invited to attend an information session (about 1 hour) and to provide written informed consent to take part in the research.<br><br>Participants in the actigraphy study were required to be non-smokers in good general health with a body mass index between 18.5-27.0 kg/m2. Individuals were ineligible if they reported shift work (paid work between 23:00 and 07:00) or if they planned on traveling across time zones during the study. Self-selection bias may be present because students had to decide whether to participate in the research. However, it is unlikely that self-selection bias had a meaningful impact on the results because the actigraphy data supported/verified the findings that were based on university-archived data.
Ethics oversight	Analyses of university-archived student data were approved by the NUS Learning Analytics Committee on Ethics (LACE).<br><br>Research procedures in the actigraphy study were approved by the NUS Institutional Review Board.

Note that full information on the approval of the study protocol must also be provided in the manuscript.
